# Engineered extracellular vesicles mediated CRISPR-induced deficiency of IQGAP1/FOXM1 reverses sorafenib resistance in HCC by suppressing cancer stem cells

**DOI:** 10.1186/s12951-023-01902-6

**Published:** 2023-05-18

**Authors:** Cong He, Doulathunnisa Jaffar Ali, Yuhua Qi, Yumin Li, Beicheng Sun, Rui Liu, Bo Sun, Zhongdang Xiao

**Affiliations:** 1 State Key Laboratory of Bioelectronics, School of Biological Science and Medical Engineering, Southeast University, Nanjing, 210096 Jiangsu China; 2 Department of Hepatobiliary Surgery, Nanjing Drum Tower Hospital, The Affiliated Hospital of Nanjing University Medical School, Nanjing, 210008 Jiangsu China; 3 NHC Key Laboratory of Enteric Pathogenic Microbiology, Jiangsu Provincial Center for Disease Control and Prevention, Nanjing, 210009 Jiangsu China; 4 Department of Hepatobiliary Surgery, The First Affiliated Hospital of Anhui Medical University, Hefei, 230022 Anhui China; 5 Department of Genetic Engineering, College of Natural Science, University of Suwon, Kyunggi-Do, 445-743 Republic of Korea

**Keywords:** Extracellular vesicles, CRISPR/Cas9, liver cancer, sorafenib resistance, cancer stem cells

## Abstract

**Background:**

Sorafenib resistance poses therapeutic challenges in HCC treatment, in which cancer stem cells (CSCs) plays a crucial role. CRISPR/Cas9 can be utilized as a potential technique to overcome the drug resistance. However, a safe, efficient and target specific delivery of this platform remains challenging. Extracellular vesicles (EVs), the active components of cell to cell communication, hold promising benefits as delivery platform.

**Results:**

Herein we report the normal epithelial cell –derived EVs engineered with HN3(HLC9-EVs) show competing tumor targeting ability. Anchoring HN3 to the membrane of the EVs through LAMP2, drastically increased the specific homing of HLC9-EVs to GPC3^+^Huh-7 cancer cells rather than co-cultured GPC3^−^LO2 cells. Combination therapy of HCC with sorafenib and HLC9-EVs containing sgIF to silence IQGAP1 (protein responsible for reactivation of Akt/PI3K signaling in sorafenib resistance) and FOXM1 (self-renewal transcription factor in CSCs attributed to sorafenib resistance), exhibited effective synergistic anti-cancer effect both in vitro and in vivo. Our results also showed that disruption of IQGAP1/FOXM1 resulted in the reduction of CD133^+^ population that contribute to the stemness of liver cancer cells.

**Conclusion:**

By reversing sorafenib resistance using combination therapeutic approach with engineered EVs encapsulated CRISPR/Cas9 and sorafenib, our study foreshadows a path for a better, accurate, reliable and successful anti-cancer therapy in the future.

**Graphical Abstract:**

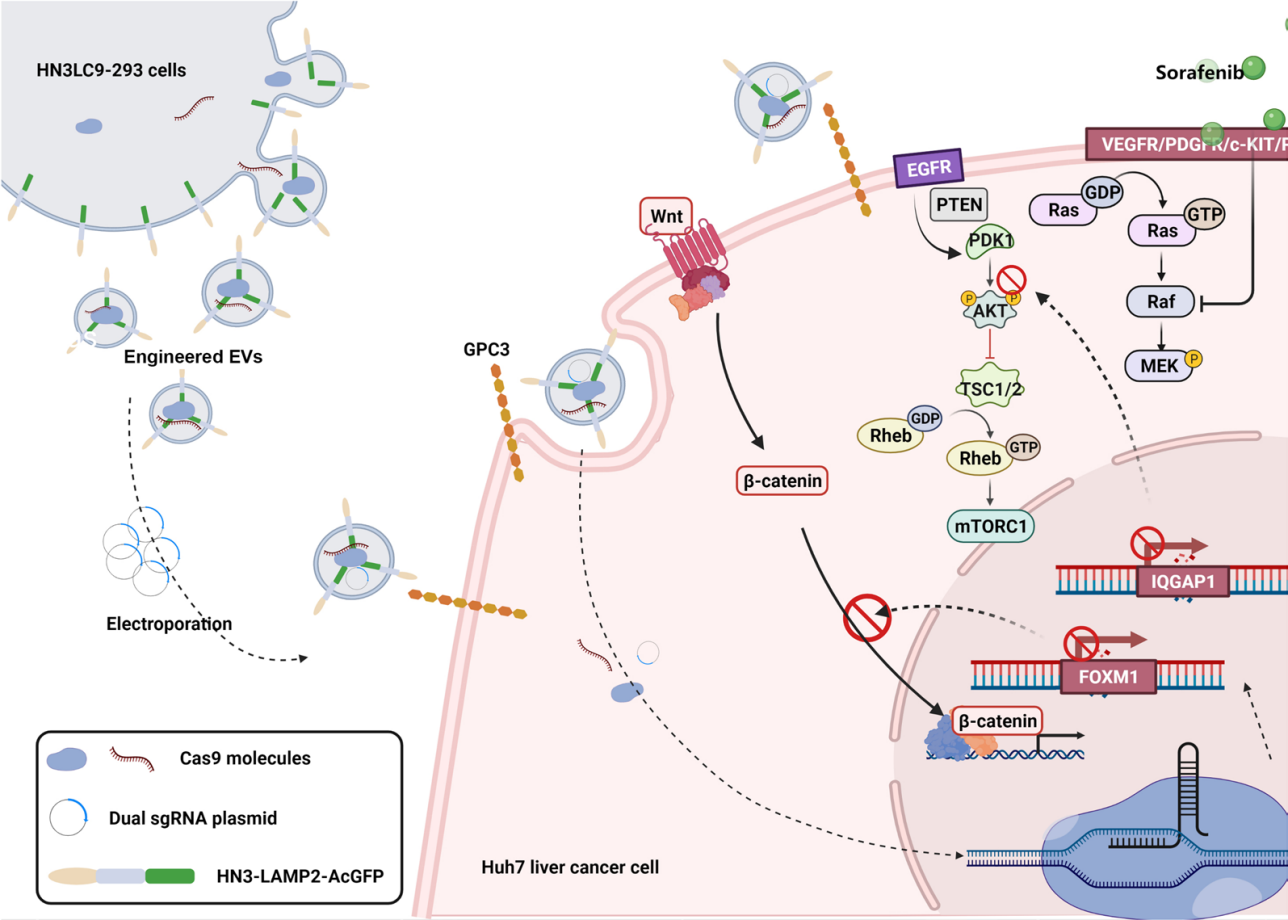

**Supplementary Information:**

The online version contains supplementary material available at 10.1186/s12951-023-01902-6.

## Background

Hepatocellular Carcinoma (HCC) is the second leading cause of cancer-related death [1, 2]. Sorafenib is an FDA approved drug used clinically to treat advanced hepatocellular carcinoma [3, 4]. However, reported resistance to this drug constrains successful liver cancer therapy [5–7]. Accumulating evidence pointed out that cancer stem cells (CSCs), with verified stemness markers (eg. CD133, EpCAM, CD90, ALDH) [8, 9], might induce sorafenib resistance in HCC [10, 11] and are considered as novel therapeutic targets [12, 13].

CRISPR/Cas9, the powerful genome editing tool, enables efficient genetic aberrations correction. Especially, it can be used for target oncogenes and chemo-resistant genes and could be utilized as a potential cancer therapeutic approach. However, safe, efficient and target- specific delivery of this genome editing tool is an impediment in effective clinical applications. Although, high loading viral vectors are currently used in multiple gene/drug delivery applications, safety remains a major concern to consider them for clinical therapies [14–16].

Extracellular vesicles (EVs), are naturally secreted lipid bilayer covered nano-vesicles by several cell types. They transport functional bio-molecules and play an immense role in intercellular communication. So they are considered as potential promising delivery vehicles for clinical applications in recent years [17, 18]. Extensive studies revealed that the modification of EVs enables them to transport cargoes to specifically target cells. Our recent study proved that fusion of HN3(an excellent affiliative antibody for GPC3 [19, 20]) with exosomes derived from epithelial cells could advance liver cancer cell targeting specificity and thus could positively enhance the anti-cancer effect of sorafenib on GPC3 over expressed liver cancer cells [21].

IQ-domain GTPase-activating proteins (IQGAPs) and Forkhead Box M1(FOXM1) are two major proteins frequently studied in cancer therapies. As a scaffold protein, IQGAP1 is one of the key components involved in PI3K/Akt signaling pathway whose reactivation results in sorafenib resistance [22–24]. In addition, overexpression of FOXM1 in HCC contributes to upregulation of wnt signaling by promoting localization of β- catenin [25, 26], which is pivotal in CSCs stemness maintenance.

Therefore, in this study, we developed middle-sized EVs [27] as delivery vehicle for CRISPR-Cas9 to reverse the therapy resistance of sorafenib by targeting these key genes and cancer stem cells. Engineered HLC9-EVs encapsulated sgIF, contributed to the suppression of IQGAP1/FOXM1and subsequent significant reduction of CD133^+^ liver cancer stem cells. Moreover, synergistic anti-tumor efficacy was achieved by our engineered EVs when combined with sorafenib treatment. Taken together, our engineered EVs showed effective anti-tumor efficacy in HCC and CSCs over sorafenib resistance for prospective clinical applications.

## Results

### CD133^+^ Huh7 are less responsive to sorafenib treatment

**Fig. 1:**
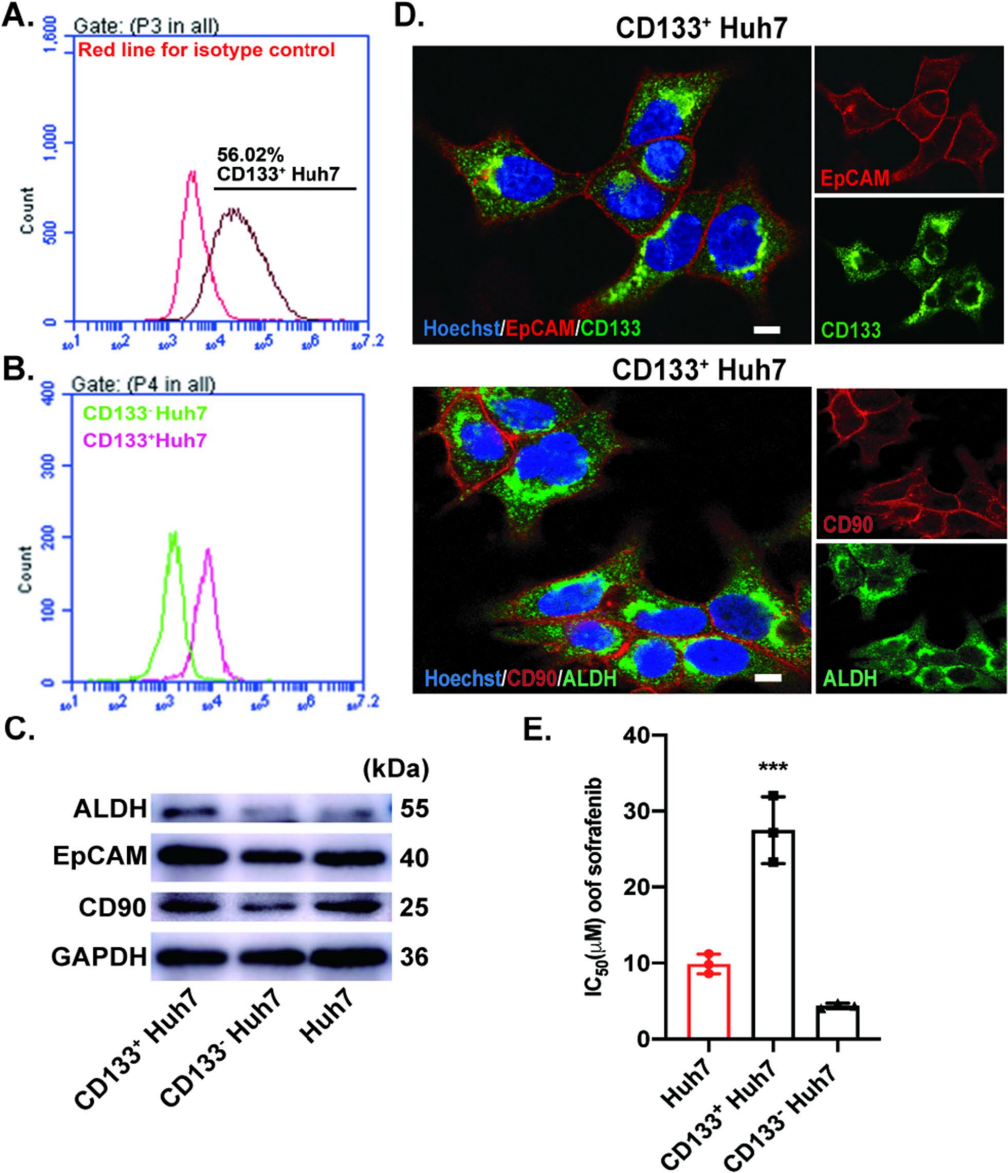
CD133^+^ Huh7 cell population contributes to sorafenib resistance. **A**, **B** CD133 positive Huh7 cells were sorted and verified with Flowcytometry. **C** The expression of liver cancer stem cell markers (ALDH/ EpCAM/ CD90) was examined in sorted and unsorted Huh7 cells by western blotting. **D** The expression of CSCs markers (CD133/EpCAM/ ALDH/ CD90) was analyzed using immunostaining. Scale bar: 1 µm. **E** Different concentration of sorafenib was used to treat CD133^+^ Huh7, CD133^−^ Huh7 and Huh7 cells, the IC_50_ value was analyzed with CCK-8 assay. Huh7 cells was set as control. Data are expressed as mean ± SD. n = 3; ***p < 0.001 by student’s t-test

Recent cancer stem cell (CSCs) theory provides a new prospect of tumor initiation and propagation [8, 9]. In HCC, CSCs were identified to be resistant to sorafenib treatment [28] and, more importantly CD133 was reported as a crucial marker of these cells [29, 30]. Thus, to identify the role of CD133 in HCC sorafenib resistance, CD133^+^ Huh7 and CD133^−^ Huh7 cells were sorted out using CD133-conjugated microbeads. As shown in Fig. 1A, the CD133^+^ (56.02%) cells were sorted, and the two populations of the cells were verified via FACS (Fig. 1B). To verify the stemness of these cells, the expression of liver cancer stem cell markers EpCAM, ALDH and CD90 were examined in CD133^+^ Huh7, CD133^−^ Huh7 and Huh7 cells. The western blot analysis showed that there was a significantly increased expression of ALDH in sorted CD133^+^ Huh7 cells compared to CD133^−^ Huh7 and Huh7 cells, along with a considerable increased expression of CD90 and EpCAM (Fig. 1C). In addition, immunofluorescence staining with above mentioned liver cancer stem cells markers was performed with sorted CD133^+^ Huh7 cells which confirmed that sorted cells are purely enriched liver cancer stem cells (Fig. 1D). CCK-8 assay was then assessed to investigate the half maximal inhibitory concentration (IC_50_) of sorafenib in CD133^+^ Huh7, CD133^−^ Huh7 and Huh7 cells which is found to be significantly high in CD133^+^ cells (27.15 µM) than CD133^−^ cells (4.35 µM). The results exhibited that CD133^+^ Huh7 cells were less responsive to sorafenib compared to the other two cells (Fig. 1E). Thus, in consistent with the previously published results, our data also revealed that CD133^+^ Huh7 cells contributes to sorafenib resistance in HCC.

### Engnineered HLC9-EVs confers specific liver cancer cell targeting

**Fig. 2:**
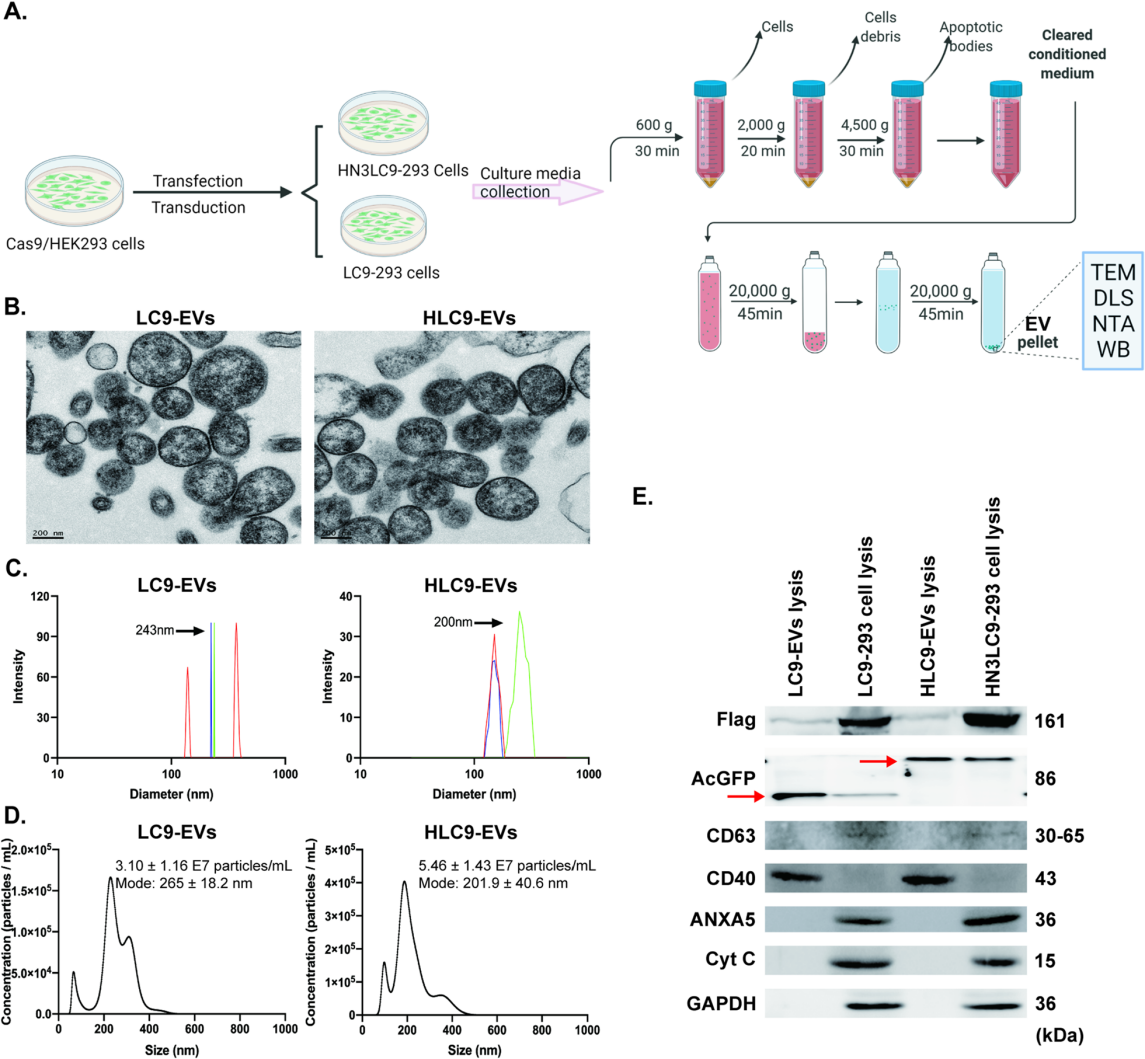
Characterization of engineered EVs. **A** Schematic diagram for isolation procedure of engineered EVs. **B** TEM, **C** DLS and **D** NTA analysis showed the morphology, size distribution and particle concentration of engineered LC9-EVs and HLC9-EVs. **E** Western blotting analysis of CD40, Flag and AcGFP (red arrows) in both engineered cells and its produces EVs. CD63 and ANXA5 have been used to exclude the presence of exosomes (small-sized EVs) and apoptotic antibodies (large-sized EVs) while Cytochrome C (Cyt C) has been used as the negative marker of EVs

HN3 refers a human antibody, which was reported to have excellent affinity to overexpressed GPC3 protein on the surface of liver cancer cells in several CAR-T studies [19, 20]. Pure engineered EVs donor cell lines with the stable expression of HN3-LAMP2-AcGFP/Cas9 and LAMP2-AcGFP/Cas9 were generated as previously reported [21]. For good encapsulation, here we used middle-sized EVs, which are identified as microvesicles. The EVs produced by HN3LC9-293 and LC9-293 cells were isolated and purified through ultracentrifuge method (Fig. 2A) as explained in methods section and henceforth denoted as HLC9-EVs and LC9-EVs, respectively. Characterization of EVs with TEM showed that both the EVs were membrane surrounded nano-vesicles and were round in shape with the particle size of 100 to 400 nm (Fig. 2B). Size distribution with DLS and NTA analysis showed that the EVs were approximately 200 to 300 nm in size with 3.10–5.46 × 10^7^ particles/mL (Fig. 2C, D). Further, both engineered LC9-EVs and HLC9-EVs were enriched for CD40, AcGFP (to identify HN3-LAMP2-AcGFP/LAMP2-AcGFP fusion protein) and Flag (fused with Cas9) markers, the western blot results from Fig. 2E, indicated the successful incorporation of LAMP2/HN3 or LAMP2 fusion protein and Cas9 protein in the respective donor cells. Additionally, *in-vitro* and in vivo safety evaluation have also been employed to check the immunogenicity of those EVs which confirmed that the isolated EVs are less immunogenic in nature (Additional file 1: Figure S2).

In continuation to the characterization, cellular internalization was investigated to confront the delivering abilities of the obtained engineered EVs. Delivering of EVs content to desired recipient cells is the crucial point while considering the suitable vehicles. Thus, cellular internalization of DiD –labeled LC9-EVs and HLC9-EVs at 3 h was examined with confocal microscopy and FACS. As shown in the confocal images (Additional file 1: Figure S3A), HLC9-EVs were competently internalized by the recipient Huh7 cells than the LC9-EVs. Also, FACS analysis indicated enhanced fluorescence signals (DiD) in Huh7 cells treated with HLC9-EVs compared to LC9-EVs, which further endorsed the confocal results (Additional file 1: Figure S3B). Interestingly, an intensive absorption of HLC9-EVs was observed in CD133^+^ Huh7 cells compared to the CD133^−^ Huh7 and Huh7 cells (Additional file 1: Figure S3C). Irrespective of the similar particle size (Fig. 2B), the quicker targeting ability of HLC9-EVs might be due to the presence of HN3 which specifically target the GPC3 that is overexpressed on Huh7 cells (Additional file 1: Figure S3D).

**Fig. 3:**
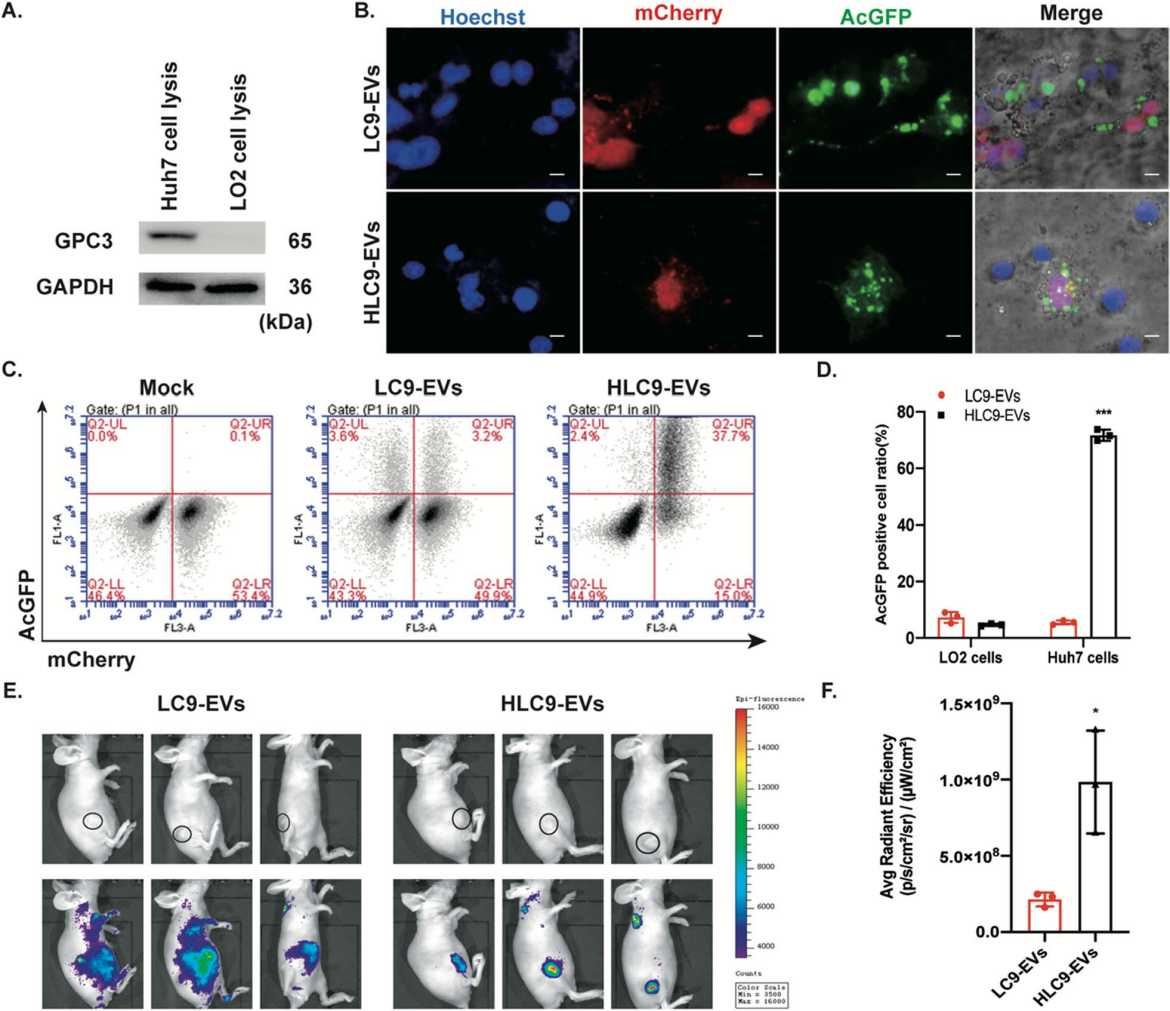
EVs-mediated liver cancer cell specific targeting. **A** Western blotting analysis of the presence of GPC3 expression in Huh-7 and LO2 cells. **B** In co-culture model, GPC3-mediated liver cancer cell specific targeting of EVs at 3 h post treatment. Red represents GPC3^+^ mcherryHuh-7 cells. Green represents LC9-EVs and HLC9-EVs. Scale bars: 1 µm. **C**, **D** FACS analysis of co-cultured cells post EVs treatment and quantitative evaluation respectively. LC9-EVs was used as control. Data are expressed as mean ± SD. n = 3; ***p < 0.001 by student’s t-test. **E** In vivo biodistribution of DiD-labeled EVs administrated intravenously in Huh7 xenograft mice and **F** corresponding fluorescent intensity quantification at tumor sites. LC9-EVs was used as control. Data are expressed as mean ± SD. n = 3; *p < 0.05 by student’s t-test

To further authorize the targeting specificity of engineered HLC9-EVs, a co-culture model was established using GPC3^+^ mcherryHuh7 and GPC3^−^ LO2 cells. LC9-EVs and HLC9-EVs were separately added to the co-cultured cells for 3 h and checked for their GPC3^+^ target specific internalization. As expected, HLC9-EVs were taken up by GPC3^+^ mcherryHuh7 cells more selectively and efficiently than by the GPC3^−^ LO2 cells (Fig. 3A，B). On the other hand, there was no target tropism towards GPC3^+^ mcherryHuh7 cells for LC9-EVs. In addition, cancer cell specific targeting of HLC9-EVs was quantified using FACS (Fig. 3C, D). Exposure of co-cultured cells to HLC9-EVs, resulted in 71.7% of AcGFP positive GPC3^+^ mcherryHuh7 cells while only 5.5% LO2 cells acquired AcGFP signal. Conversely there was not much difference in the percentage of AcGFP positive signals in GPC3^+^ mcherryHuh7 and GPC3^−^ LO2 cells (4.7% and 7.3%, respectively) when the co-culture model was incubated with control LC9-EVs. These data indicate that HN3 antibody bearing HLC9-EVs binds efficiently to the extracellular region of GPC3 expressed on the surface of Huh7 cells and thus conferring enhanced specific liver cancer cell targeting ability compared to control LC9-EVs.

The tumor targeting competence of these EVs were further evaluated in vivo through an IVIS fluorescence imaging system. DiD-labeled LC9-EVs and HLC9-EVs were administrated intravenously to Huh7 xenograft mice and the fluorescent signals were observed 6 h post-injection. Similar to the above mentioned in vitro cellular internalization results, significant accumulation of fluorescent- labeled HLC9-EVs was observed in specific tumor sites in vivo (Fig. 3E, F) than LC9-EVs. Though some DiD –labeled LC9-EVs were found in tumors, they were scattered in other tissues which indicated that HLC9-EVs are more tumor specific and efficiently target liver cancer cells than LC9-EVs.

### HLC9-EVs function as effectual natural vehicles

**Fig. 4:**
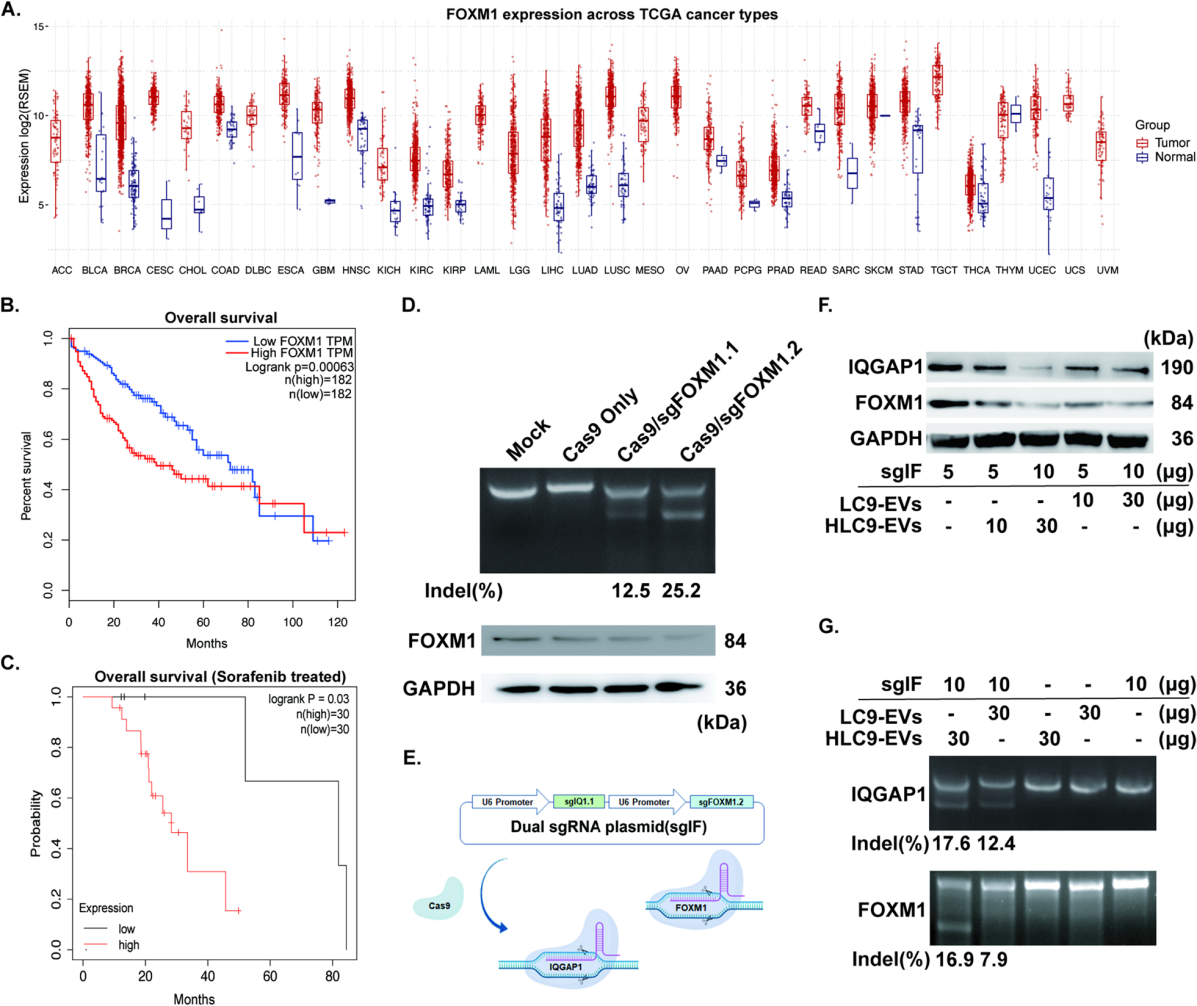
FOXM1 and EVs -mediated gene editing. **A** Analysis of FOXM1 expression in HCC tumor and normal tissue using TCGA database. **B** Overall survival (OS) curves of HCC patients and **C** sorafenib treated HCC patients. **D** Cas9:sgRNA-mediated indels were assessed by T7E1 assay and corresponding protein inhibition level was evaluated through western blotting. **E** Schema of dual sgRNA plasmid induced cleavage and **F** reduction of targeted gene expression level was analyzed using different quantity combination of EVs and sgRNA by western blot along with **G** related indels were examined using T7E1 assay

Next, as the core of this study, engineered EVs were investigated for being used as prompt delivery vehicles for CRISPR/Cas9 in HCC treatment. According to TCGA database, FOXM1 showed notable differences between tumor and normal tissue in liver cancer (Fig. 4A). Kaplan–Meier survival analysis revealed the overexpression of FOXM1 was significantly related with the poor prognosis of HCC patients (Fig. 4B), especially in sorafenib treated HCC patients (Fig. 4C). After various consideration of human genome locus of FOXM1, two sgRNAs were designed against two different sites of FOXM1 and named as sgFOXM 1.1 and sgFOXM 1.2 (Additional file 1: Table S1). Cleavage efficiency of designed sgRNAs at particular targeted site was analyzed by T7E1 assay. Compared to Cas9/sgFOXM1.1, Cas9/sgFOXM1.2 transfected Huh7 cells acquired more efficient cleavage (25.2%) at the targeted site and no cleaved fragments were detected in Cas9/sgRNA^−^ treated control cells (Fig. 4D). This was simultaneously verified at protein level by western blot (Fig. 4D) in which Cas9/sgFOXM1.2 transfection resulted in much effective lessening of protein expression in Huh7 cells. Hence, sgFOXM1.2 alone was used further in this study. Besides, sgIQ 1.1 was also chosen to be used in the dual sgRNA vector system along with the sgFOXM1.2, as it was found to be effective against in our previous studies [31].

Furthermore, to examine the efficiency of engineered HLC9-EVs as a potential delivery platform, HLC9-EVs were loaded with dual sgRNA (Fig. 4E, sgIQ 1.1 + sgFOXM 1.2, henceforth mentioned as sgIF) via electroporation [32]. As shown in Fig. 4F, various concentrations of EVs and sgIF plasmids were tested to identify the optimal one in which a maximum reduction of IQGAP1 and FOXM1 could be attained. Later, to find the gene editing efficiency of sgIF loaded EVs, in vitro T7E1 assay was performed. Intriguingly, 17.6% and 16.9% indels were found (Fig. 4G) when sgIF and engineered EVs (10: 30 µg) were electroporated at approximately 5.5% loading efficiency (Additional file 1: Figure S4). These results showed that HLC9-EVs could competently transport the encapsulated DNA into the recipient liver cancer cells via HN3-GPC3 mediated targeted delivery and achieve considerable cleavage, which could be considered as effective natural vehicles for CRISPR system.

### sgIF loaded HLC9-EVs with sorafenib exhibits synergystic anti-cancer effect

**Fig. 5:**
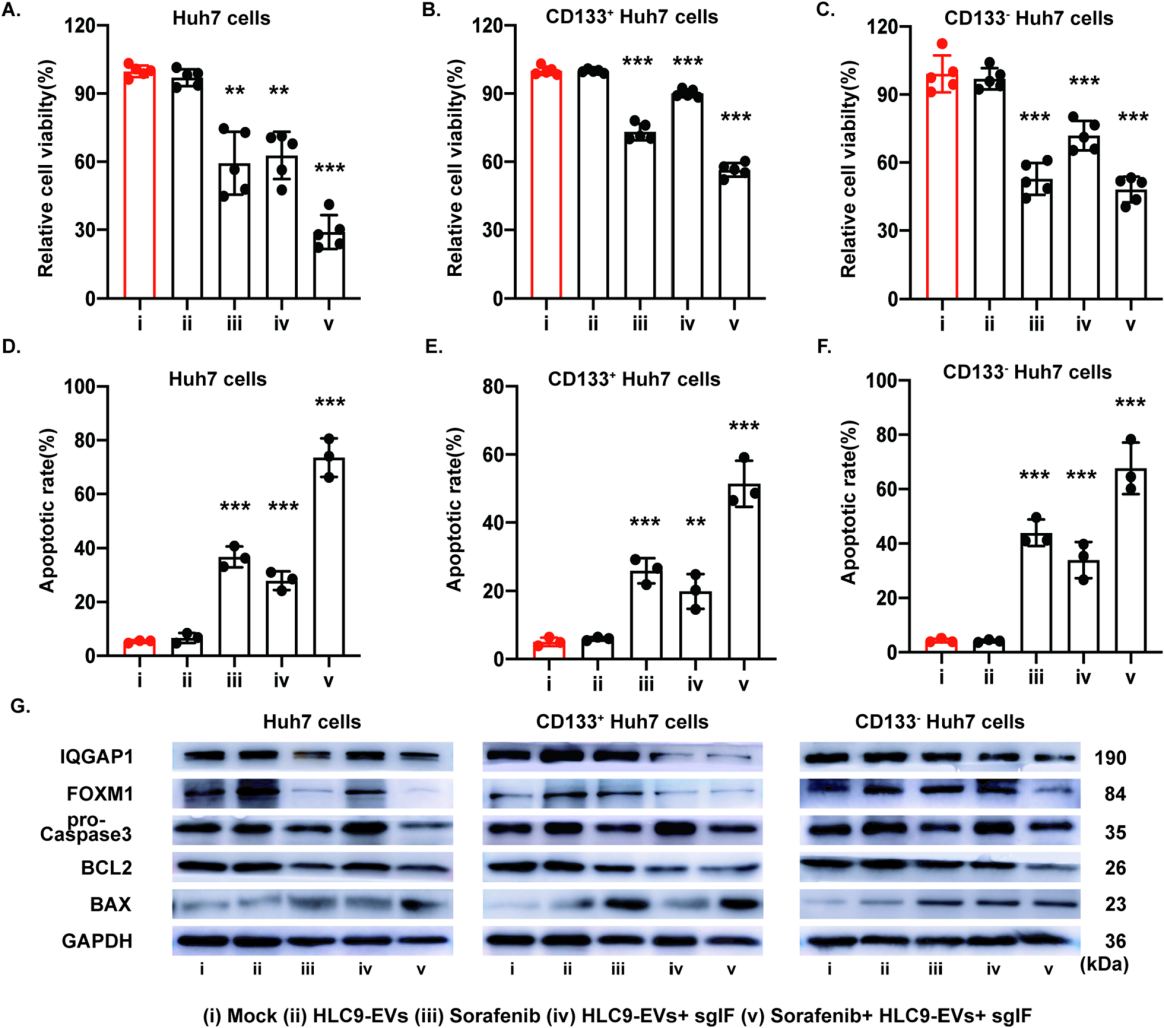
Combined therapy of EVs -mediated IQGAP1/FOXM1 destruction with sorafenib. **A**–**C** CCK-8 analysis of CD133^+^ Huh7, CD133^−^ Huh7 and Huh7 cells.10 μM concentration of sorafenib was used. n = 5. **D**–**F** Corresponding apoptotic rate was evaluated with FACS by using Annexin V/PI staining kit. **D** Differential protein expression level of target gene IQGAP1/FOXM1 along with apoptosis markers (pro-Caspase3, BCL2 and BAX) were examined. GAPDH was used as an internal control. Mock group was set as control, data are expressed as mean ± SD. n = 3; *p < 0.05, ***p < 0.001, by student’s t-test

Combination therapy often upgrades the effectiveness of cancer therapeutics by its promising synergistic effects [32]. Sorafenib, a remarkable anti-tumor drug used in various types of cancers including HCC [33, 34]. Hence, the following study was conducted to find the synergistic effect of sgIF loaded HLC9-EVs when combined with sorafenib drug in CD133^+^ Huh7, CD133^−^ Huh7 and Huh7 cells. CCK-8 assay indicated that sgIF loaded HLC9-EVs plus sorafenib competently slowed down unsorted Huh7 cells propagation and thus contributed to more synergistic anti-proliferative effect (71.0% ± 7.4%) than sorafenib (40.5% ± 13.8%) and sgIF loaded HLC9-EVs(37.3% ± 10.3%) alone treatment (Fig. 5A). Astonishingly, sgIF loaded HLC9-EVs in combination with sorafenib drug exhibited a noteworthy anti-proliferative effect (43.7% ± 3.0%) on CD133^+^ Huh7 cells, which was in separate 10 µM sorafenib drug (26.9% ± 3.6%) and sgIF loaded HLC9-EVs (9.9% ± 1.7%) treatments (Fig. 5B). On the other hand, there is no significant difference in the anti-proliferative effect of sgIF + HLC9-EVs + sorafenib (51.9% ± 5.6%) and sorafenib (47.2% ± 6.9%) alone treatment group in CD133^−^ Huh7 cells as they are sorafenib sensitive (Fig. 5C).

Added, synergistic pro-apoptotic effect was evaluated by FITC-annexin V/ PI kit (Additional file 1: Figure S5). As given in Fig. 5D–F, the percentage of apoptotic cell rate was in consistence with above proliferation results. Interestingly, considerable elevated apoptotic rate was found in sgIF + HLC9-EVs + sorafenib group in all CD133^+^ Huh7, CD133^−^ Huh7 and Huh7 cells. Furthermore, this was endorsed by the reduced expression of proapoptotic markers BCL2 as well as enhanced expression levels of BAX using western blot (Fig. 5G). The inhibition of protein expression of IQGAP1 and FOXM1 were also checked simultaneously in the same cells to confirm that the changes in the apoptotic markers are the results of destruction of IQGAP1 and FOXM1. The outcome verified that the destruction of IQGAP1 and FOXM1 with HLC9-EVs encapsulated sgIF plus sorafenib could promote efficient apoptosis in Huh-7 cells (Fig. 5G). Remarkably, the spectacular synergistic effect was observed in CD133^+^ Huh7 and Huh7 cells but not in CD133^−^ Huh7 cells, which indicated the dual inhibition of IQGAP1 and FOXM1 by engineered HLC9-EVs might regulate CD133^+^ population to make it more responsive to sorafenib drug. Taken together, these results strongly evidenced that sgIF loaded HLC9-EVs showed competent tumor killing ability, enhanced in combination with sorafenib, which contributes to thriving synergistic anti-cancer effect.

### Knock-out of IQGAP1/FOXM1 results in reduction of CD133^+^ population and reverses sorafenib resistance

**Fig. 6:**
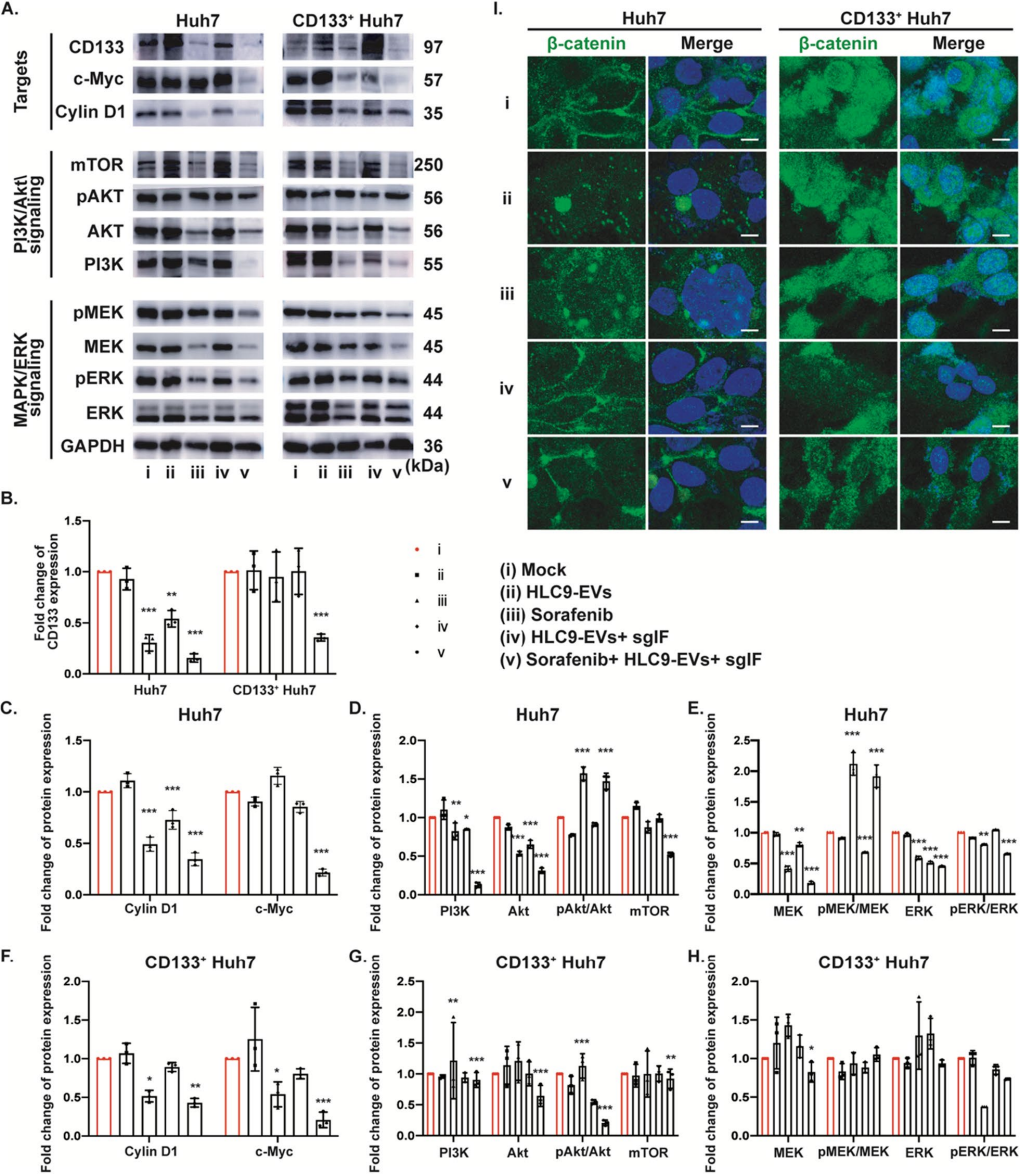
Knockout of IQGAP1/FOXM1 upgrades sorafenib therapeutic effect. **A** PI3K/Akt and MAPK/ERK signaling related protein expression level were examined using western blotting and **B**–**H** corresponding quantitative protein expression results. **I** Nuclear localization of β-catenin was analyzed with immunostaining. Scale bar: 1 μm. Mock group was used as control. Data are expressed as mean ± SD. n = 3; *p < 0.05, ***p < 0.001, by two-way ANOVA

IQGAP1 is a scaffold protein which facilitates the interaction of mTOR and Akt and thus promotes liver cancer progression [35]. Activation of Akt signaling results in sorafenib resistance [24]. Sorafenib is mainly acting on targeting Raf/MAPK/ERK pathway [34]. Accordingly, to uncover the knock-out of IQGAP1/FOXM1 could make changes in the Akt and MAPK signaling pathways, the expression level of Akt, pAkt, PI3K, mTOR, MEK, pMEK, ERK, pERK, c-Myc and Cyclin D1 was examined in Huh7, CD133^+^ Huh7 and CD133^−^ Huh7 (Additional file 1: Figure S6) cells. Western blot analysis revealed that the combined therapy of sgIF loaded HLC9-EVs plus sorafenib decreased the Akt, PI3K, mTOR, MEK and also their downstream transcriptional factors cyclin D1 and c-Myc (Fig. 6A, C–G). However, in both Huh7 and CD133^+^ Huh7 cells, sorafenib treatment brought increase of the phosphorated form(pAkt)/Akt, which indicated the reactivation of Akt signaling, whereas the sgIF + HLC9-EVs + sorafenib reverse the activation, especially in CD133^+^ Huh7 cells (Fig. 6A, D and G). In Huh7 cells, the expression level of total MEK and ERK was significantly downregulated by sgIF + HLC9-EVs + sorafenib (Fig. 6E). At the same time, the activation of MAPK signaling (pMEK/MEK and pERK/ERK) was downregulated too. In CD133^+^ Huh7 cells, the total MEK and ERK level increased with sorafenib treatment and conversely reversed by sgIF + HLC9-EVs + sorafenib (Fig. 6H). Taken together, the sgIF loaded HLC9-EVs effectively inhibits PI3K/Akt and MAPK/ERK, the two important signaling pathways that actively contribute to cancer progression and annul sorafenib resistance.

Wnt/β-catenin signaling is essential for CSCs regulation and tumorigenesis [36]. IQGAP1 promotes nuclear translocation of β-catenin in HepG2 cells, involving in Wnt signaling pathway [37]. FOXM1 interacts with β-catenin and contributes to further β-catenin nuclear localization in glioma tumorigenesis [25]. Thus, the possibility of IQGAP1/FOXM1 knock-out to reduce CD133^+^ population and reverse sorafenib resistance in vitro by sgIF loaded HLC9-EVs plus sorafenib was next to investigated. Huh7 and CD133^+^ Huh7 cells were treated with sgIF loaded HLC9-EVs plus sorafenib for 48 h followed by immunostained with fluorescently labeled β-catenin (Fig. 6I). As anticipated, sgIF loaded HLC9-EVs plus sorafenib treatment effectively inhibited β-catenin nuclear translocation in both Huh7 and CD133^+^ Huh7 cells compared to untreated control. In addition, to our great surprise, expression of CD133 marker was also reduced much in the sgIF loaded HLC9-EVs plus sorafenib treatment compared to sorafenib alone treatment (Fig. 6A, B). Altogether, these results, strongly confirm that knock-out of IQGAP1/FOXM1 with sgIF loaded HLC9-EVs + sorafenib in effect reduces CD133^+^ CSCs and reverses sorafenib resistance, and hence stands as promising novel therapeutic approach for thriving liver cancer treatments in the future.

### sgIF loaded HLC9-EVs synergizes in vivo anti-tumor efficacy of sorafenib

**Fig. 7:**
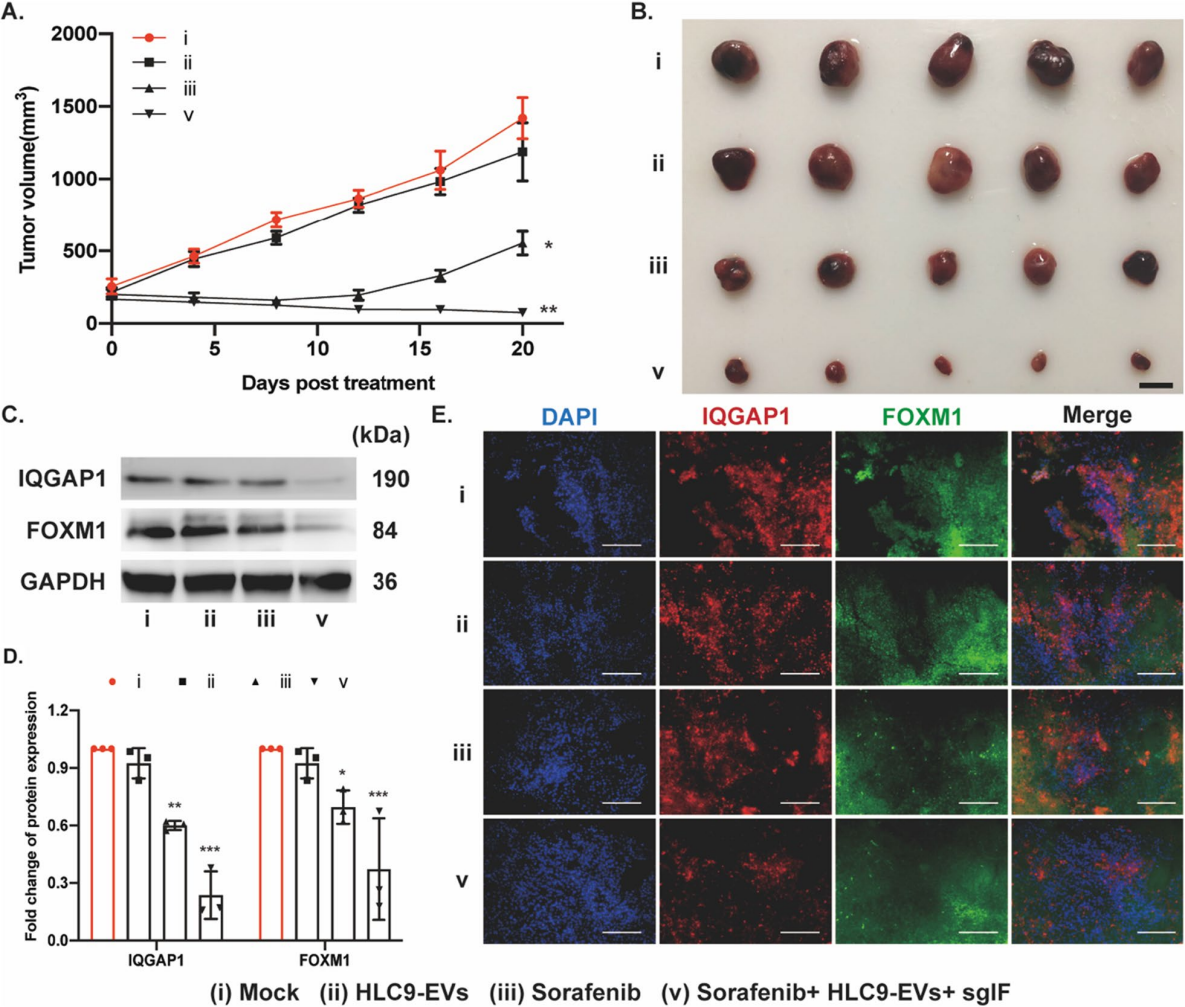
Anti-cancer effects of sgIF loaded HLC9-EVs in combination with sorafenib treatment in Huh7 xenografts. **A** Tumor sizes were measured once in 4 days post the initial injection. Group Mock was used as control. Data are expressed as mean ± SD. n = 5; *p < 0.05, **p < 0.01 by student’s t-test. **B** Display of excised tumors on day 21 post the initial treatment. Scale bar: 1 cm. n = 5. **C** Western blotting analysis of IQGAP1/FOXM1 expression and **D** relative protein expression level was quantified. **E** Immunofluorescence staining of IQGAP1/FOXM1 in excised tumor samples. Scale bar: 100 μm. Group Mock was used as control. Data are expressed as mean ± SD. n = 3; *p < 0.05, ***p < 0.001 by two-way ANOVA

After evidencing flourishing synergistic anti-tumor effect in vitro, sgIF loaded HLC9-EVs plus sorafenib was then tested for its in vivo synergistic anti-tumor efficacy. Mice bearing Huh7 xenograft were set as the model to study the in vivo anti-tumor curative effects of sgIF loaded HLC9-EVs. When the size of the tumor reached approximately 0.2 cm^3^, HLC9-EVs encapsulated sgIF along with sorafenib was injected to mice twice with an interval of 3 days. HLC9-EVs without any sgIF encapsulation and sorafenib also administered intravenously with the same interval as control. As predicted, the intravenous injection of sgIF loaded HLC9-EVs plus intraperitoneal sorafenib injection reduced tumor size much more effectively than the independent HLC9-EVs and sorafenib treatments (Fig. 7A, B). Further, in the final stage of the experiment, mice were sacrificed and the protein expression level of IQGAP1 and FOXM1 in the excised tumor samples was analyzed using western blotting. In consistent with the previous in vitro experimental results, sgIF loaded HLC9-EVs plus sorafenib treatment much effectively suppressed the expression of IQGAP1 and FOXM1 within xenografted tumors (Fig. 7C). The inhibition of IQGAP1 and FOXM1 in the tumors was simultaneously verified with immunostaining assay (Fig. 7D). Altogether, the in vivo results confirmed that sgIF loaded HLC9-EVs could deliver Cas9 and sgIF to the targeted tumor site and show remarkable anti-tumor effects with sorafenib drug. Therefore, our results attested that HN3 fused normal HEK293 cells derived EVs is good at targeting and homing at tumor sites and thus could be a good choice for safe and target specific delivery vehicles. By suppression of IQGAP1/FOXM1to synergize sorafenib treatment, engineered EVs provides a new approach for combination therapy in HCC.

## Discussion

Sorafenib is highly recommended drug for treating HCC at present, yet sorafenib resistance is reported increasingly [38, 39]. Cancer stem cells represent a rare subpopulation of cells within the tumor and are considered as pivotal as they render resistance to conventional cancer therapies [40, 41] and also are difficult to precisely eradicate due to their heterogeneity and plasticity. Amongst few important CSC markers, CD133 is considerably interesting as they actively participate in Wnt/β-catenin signaling [42], a significant pathway in the maintenance of CSCs [43, 44]. In this study, we sorted CD133^±^ Huh7 cells with CD133-conjugated microbeads. Both sorted and unsorted cells were treated with sorafenib and subjected to CCK-8 assay in which CD133^+^ Huh7 showed less cytotoxic/cell death response to sorafenib than CD133^−^Huh7 and Huh7 cells which verified that CD133^+^ Huh7 population could contribute to sorafenib resistance in HCC.

CRISPR genome editing system enables accurate gene modification at desired genomic locus and has been identified as a revolutionary tool for cancer therapy [45]. Yet, lack of safe, efficient and precise delivery vehicle limits its effective implement in clinical therapy. Irrespective of the availability of high loading viral vectors [46, 47], biomimetic nanocarrier systems including cell membranes, live cells and EVs gathered more interest in recent gene/drug delivery investigations as they might be safer method [48]. Drug leakage is very common in living cell -based drug delivery system whereas cell storage also needs to considered. Moreover, the cell membrane extraction process might impair their targeting ability [49, 50]. Hence, in this study we engineered natural secreted EVs for targeted CRISPR delivery. More importantly, the yield of EVs is another issue to be considered while using EVs as delivery vehicles. To overcome this, researchers have utilized external stimulations such as hypoxia and physical method to improve the production of extracellular vesicles [51]. Also, another interesting report claimed that specific gene modification could help for the continuous secretion of extracellular vesicles [52]. So, the proper methodology needs to be further investigated in depth to upgrade the yield of EVs for them to be utilized in large-scale animal studies. In our current work, the donor cells have been cultured in large scales to get enough amount of EVs to perform the relevant studies. In addition, the complex composition of EVs limits its further therapeutic application, while the subsequent genomic and proteomic analysis remain to be studied. Standardized EVs from HEK293 cells might be a way to solve this problem. So, in our study we used HEK293-LC9 cells to generate these EVs.

Our previous work has shown that fusion of HN3 with engineered exosomes could increase its tumor target specificity via HN3-GPC3 axis [21]. In this study, for the purpose of enriched CRISPR delivery, we investigated the homing ability of middle sized EVs secreted by HN3 fused donor cells. TEM, DLS and NTA analysis confirmed the isolated engineered EVs are lipid bilayer covered, round shaped nano-vesicles with about 100–250 nm in particle size. Western blots authenticated the presence of marker protein CD40, HN3LAMP2/LAMP2 and Cas9 in EVs. To further characterize, both LC9-EVs and HLC9-EVs were introduced to a co-coculture model (GPC3^+^ mcherryHuh7 + GPC3^−^ LO2 cells), in which HLC9-EVs (HN3 fused EVs) specifically target GPC3^+^ cells rather than GPC3^−^cells. However, control LC9-EVs did not exhibit any specific tropism. In vivo real time imaging confirmed the competing tumor homing ability of HLC9-EVs than LC9-EVs. This supported that HN3 sequences of HLC9-EVs could be precisely recognized by the extracellular GPC3 region expressed on Huh7 cells.

Previous work by our group and other groups have shown that IQGAP1 impeded the action of sorafenib by scaffolding Akt activation [53]. Inhibition of IQGAP1 could downregulate Akt signaling [54], which might be reverse the sorafenib resistance [55]. In addition, FOXM1, a pivotal regulator of cell cycle progression, aids stemness maintenance in CSCs by supporting translocation of β-catenin to nucleus in liver cancer cells [56]. Hence, a dual sgRNA plasmid to target both IQGAP1 and FOXM1 had been constructed and incorporated into engineered EVs via electroporation, which were then incubated with Huh7 cells. Considerable cleavage efficiency was witnessed at precise sites in both IQGAP1 and FOXM1 treated with sgIF loaded HLC9-EVs. This proved that HLC9-EVs could effectively deliver the plasmid DNA via HN3-GPC3 mediated target specificity and function as effective natural CRISPR/Cas9 carrier.

Combination therapies attracted enough attention over recent years. As an approach of combination therapy, the anti-tumor effect of sgIF loaded HLC9-EVs plus sorafenib was investigated both in vitro and in vivo. sgIF loaded HLC9-EVs plus sorafenib showed upgraded congenerous anti-proliferative effect on Huh7 cells than separate sgIF loaded HLC9-EVs and sorafenib treatments. In consistent, sgIF + HLC9-EVs plus sorafenib had reached much higher apoptotic rate than the individual treatments. Further western blot analysis indicated the loss of IQGAP1 and FOXM1 and subsequent induction of apoptosis via regulation of apoptotic proteins in Huh7 cells treated with sgIF loaded HLC9-EVs plus sorafenib. Similar results were observed in sorted CD133^+^ Huh7 and CD133^−^ Huh7 cells. Combination treatment of CD133^+^ Huh7 cells with sgIF + HLC9-EVs plus sorafenib resulted in significantly high anti-proliferative and pro-apoptotic rate compared to mono treatment with sorafenib alone. Further, dual knock-out of IQGAP1/FOXM1 decreased the protein expression level of Akt, PI3K, mTOR, MEK and also their downstream transcription factors cyclin D1 and c-Myc. Interestingly, the activation of Akt was increased while treating with sorafenib in both Huh7 and CD133^+^ Huh7 cells suggesting a plausible treatment resistance in these cells, whereas the combined treatment (sgIF + HLC9-EVs + sorafenib) reversed the activation, especially in CD133^+^ Huh7 cells. Also, total MEK and ERK, which were upregulated with sorafenib treatment alone, were downregulated and resulted in sgIF + HLC9-EVs + sorafenib treated CD133^+^ Huh7 cells. Taken together, reactivation of Akt and ERK signaling with sorafenib confirmed CD133^+^ Huh7 population contribute to resistance in sorafenib treatment and the sgIF loaded HLC9-EVs effectively annulled sorafenib resistance by interfering reactivation of PI3K/Akt and MAPK/ERK, the two important signaling pathways actively involved in cancer progression. Surprisingly, dual knock-out also greatly reduced the nuclear translocation of β-catenin, a central component of Wnt signaling pathway that is essential for CSCs maintenance. This in turn affected the viability of CD133^+^ Huh7 population and hence reversed sorafenib resistance. Furthermore, treatment of sgIF loaded HLC9-EVs plus sorafenib effectively reduced the tumor size and the expression of IQGAP1 and FOXM1 in Huh7 xenografts. Taken together, these results confirms that sgIF loaded HLC9-EVs plus sorafenib is effectively targets tumor sites and exhibits potent enhanced tumor cells killing effect.

## Conclusion

To sum up, our data revealed that dual knock-out of IQGAP1 and FOXM1 using HLC9-EVs together with sorafenib administration could enhance the therapeutic effect of sorafenib on Huh7 cells by dismissing crucial signaling pathways like PI3K/Akt and MAPK/ERK. Most importantly, it also impaired the CD133^+^ cancer stem cell population which plays vital role in sorafenib resistance. Hence, this study foreshows that a better treatment approach with a combination therapy of sorafenib and CRISPR mediated gene knock out by EVs could be used in future sorafenib mediated anti-cancer treatments.

### Materials and methods

#### Cell culture

HEK-293 and Huh7 cell lines were purchased from the American Type Culture Collection (ATCC). Both cell lines were cultured in DMEM, containing 10% fetal bovine serum (FBS) and 1% of penicillin/streptomycin in a humidified incubator (37 ºC, 5% CO_2_). All cell culture reagents were obtained from Hyclone Laboratories Inc. (Logan, UT, USA). FBS was EVs-depleted by filtration with a 0.22 µm steritop filter (Millipore, USA) followed by an overnight ultracentrifugation at 110,000*g*.

EVs donor cells (LC9-293 and HN3LC9-293) expressing HN3-LAMP2-AcGFP/Cas9 and LAMP-AcGFP/Cas9(Additional file 1: Figure S1) were generated as previously reported [21]. To get CD133^+^ and CD133^−^ Huh7 cells, CD133 microbeads kit (MACS, USA) was purchased and used as per manufactures’ instruction. In brief, Huh7 cells were harvested, washed twice with 1 × DPBS, resuspended with MACS buffer, supplemented with 20 µL of CD133-conjugated microbeads, and incubated for 15 min in the refrigerator. After the incubation, the cells were washed twice with plain MACS buffer and resuspended up to 10^8 cells per 500 µL buffer. Then, the resuspended cells were flowed through the LS column placed in the MACS separator. The unlabeled cells (CD133^−^ Huh7 cells) were acquired by collecting the flow-through, and the labeled cells (CD133^+^ Huh7 cells) were flushed out by pushing the plunger into the column. Subsequently, sorted cells were stained with CD133-PE antibody (Biolegend, USA) and analyzed by Flow cytometer (BD FACSAria Fusion).

#### Immunoflurescence and confocal

CD133^+^ Huh7 cells were seeded in a 12-well plate (3 × 10^5^ cell/ well) and allowed to attach overnight. The cells were washed with 1 × DPBS, fixed using 4% paraformaldehyde, blocked by 3% BSA PBS, and then incubated overnight with anti-CD133, anti-EpCAM, anti-CD90 and anti-ALDH or beta-catenin primary antibody in 4 °C. The cells were then stained with Alexa Fluor 488 and Alexa Fluor 647 conjugated secondary antibody in 37 °C for 1 h and subsequently were counterstained with Hoechst and imaged using confocal microscopy (Leica STED, Germany).

The excised tumor samples were fixed in 4% paraformaldehyde, and paraffin- sectioned. The tumor sections were primarily deparaffinized, followed by incubation with EDTA buffer (PH 8.0) in microwave to retrieve antigens. The tumor sections were subsequently immunostained and analyzed as mentioned above.

#### Extraction and characterization of EVs

The culture supernatant of donor cells was collected and the EVs were extracted as described in our previous study [31]. Briefly, the cells were cultured in the EVs –depleted culture medium for 48 h. Later, each culture medium was harvested and sequentially centrifuged at 600*g* for 30 min, 2000*g* for 20 min and 4500*g* for 30 min at 4 °C to remove cells, debris and apoptotic bodies. The supernatants were subsequently ultracentrifuged at 20,000*g* for 45 min at 4 °C using L-80XP ultracentrifuge with Type 70 Ti rotor (Beckman Coulter, Brea, CA, USA) to obtain EVs. The pellet was resuspended in 100 μL of 1 × DPBS and was stored at − 80 °C. The EVs separated from HN3LC9-293 and LC9-293 cells were henceforth denoted as HLC9-EVs and LC9-EVs, respectively. Around 20 µL of purified EVs were lysed and analyzed with Micro BCA protein assay kit (CoWin Biotechnology, Beijing, China). The presence of markers (CD40, AcGFP and Flag) for engineered EVs were examined by western blotting.

Size distribution of EVs (LC9-EVs and HLC9-EVs) was analyzed though DLS (Zetasizer Nano ZS, Malven Instruments, UK) and NTA (NanoSight NS300, Malven Instruments, UK). Morphology of the EVs were analyzed by TEM (JEM-2100. JEOL, Japan) where purified EVs transferred onto a carbon-coated grid were kept at room temperature for 20 min and then visualized under TEM.

#### Incoporation of sgRNA plasmid

sgFOXM1.1 and sgFOXM1.2 sequences (Additional file 1: Table S1) were inserted to pCas-Guide-GFP (Origene Cat# GE100012) as previously reported [31] and turned to Cas9/sgFOXM 1.1 and Cas9/sgFOXM 1.2. The plasmids were transfected to Huh7 cells through jetPRIME reagent (Polyplus Transfection, France) following the manufacturers' protocol. After 48 h, the cells were collected and the in vitro T7E1 assay was performed. In brief, genomic DNA was extracted with Multisource Genomic DNA Miniprep Kit (Axygen, USA). With precise primers (Additional file 1: Table S1), PCR amplicons at designed target site were amplified, purified, re-annealed and followed by T7 endonuclease I (T7E1, NEB, USA) digestion. The digested DNA was then visualized by agarose gel.

To get dual sgRNA (sgIF) plasmid, plasmid B52(100708, Addgene) representing an empty plasmid backbone to express 2 sgRNAs had been purchased in which sgIQGAP1.1 (chosen based on our previous reports [21, 31]) and sgFOXM1.2 sequences were inserted by Bsmb I and Bbs I restriction site respectively according to the instruction given by the manufacturer.

The constructed sgRNA plasmid was loaded to EVs by Neon electroporation system (Thermofisher, USA) following previous protocol [21, 31]. EVs and sgIF plasmids were mixed gently in Buffer R and electroporated at 1000 mV, 10 ms and 2 pulses. After electroporation, the mixture was washed and resuspend with DPBS followed by DNase I treatment. Specific primers to amplify sgRNA plasmid were designed and utilized to verify the successful encapsulation (Additional file 1: Table S1).

#### In vitro and in vivo cellular uptake assay

The mcherry red fluorescent tag was integrated to GPC3^+^ Huh7 liver cancer cells via lenti-virus transduction and turn to be CPC3^+^ mcherry expressing Huh-7 cells. The target specific cellular uptake of EVs was examined in a co-culture model, in which equal amount of GPC3^−^ LO2 cells (3 × 10^5^) and GPC3^+^ mcherry expressing Huh-7 cells (3 × 10^5^) were seeded together in 12-well plates, to which HLC9-EVs and LC9-EVs (20ug) were added respectively and incubated for 3 h. Then the co-cultured cells were collected, washed, fixed and analyzed via flow cytometry (Accuri C6, BD, USA) and inverted fluorescence microscopy (Nikon, Japan).

Six weeks old female BALB/c nude mice were obtained from Beijing Vital River Laboratories. The establishment of Huh7 xenograft and EVs tumor targeting study were carried as previously reported [31]. In brief, purified EVs were stained with DiD (5 mM, Biotium, USA) in dark for 30 min at 37 ℃, followed by centrifugation (10, 000*g*, 15 min) for removal of the unbound dye. Later, the labeled EVs were purified and resuspended in DPBS for further use. When tumor size reached 0.1 cm^3^, DiD labeled EVs were administrated to mice intravenously and the biodistribution was observed via IVIS animal imaging system (Perkin Elmer, USA).

#### In vitro anti-tumor activity assay

Sorted and unsorted Huh7 cells were seeded in a 96 –well microtiter plates (2 × 10^4^ cells/well), to which around 30 µg of EVs loaded with 10 µg of sgIF were added with or without 10 µM sorafenib (Beyotime Technology, China). After 48 h, cell viability was assessed with CCK-8 kit (Dojindo, Janpan) by measuring the absorbance at 450 nm.

The apoptotic effect of sgIF encapsulated HLC9-EVs was analyzed with sorted and unsorted Huh7 cells by Annexin V-FITC/PI kit (Multisciences, China). Sorted and unsorted Huh7 cells were seeded in 12 -well plates (2 × 10^5 cells per well), treated with different formulations of EVs (with or without sorafenib) and incubated for 48 h. After incubation, cells were collected, washed and stained with Annexin V-FITC and PI. The stained cells were further analyzed by Accuri C6 Flow cytometer (BD Biosciences, CA) using CFlow (BD Biosciences, CA) software. Annexin V-FITC^+^ PI^−^ cluster represents early stage of apoptosis, while Annexin V-FITC^+^ PI^+^ cluster indicates cells in the late stage of apoptosis, in necrosis or dead.

#### Western blot

Huh7 cells were seeded at a density of 1 × 10^6^ cells per well in 6 –well plates and allowed to attach overnight. Then, the cells were treated with different formulations of EVs with or without sorafenib. After 48 h, total protein was extracted and quantified. Equal amounts of protein samples were separated in SDS-PAGE, transferred to a PVDF membrane, blocked and incubated with different primary antibodies (Additional file 1: Table S2), followed by HRP-conjugated secondary antibody and visualized via Tanon-4200 Chemiluminescent Imaging System. GAPDH was set as an internal control. The band intensities were analyzed by ImageJ software (NIH).

### In vivo anti-tumor activity assay

Huh7 xenografts were established as mentioned in in vitro and in vivo cellular uptake assay. When the size of tumor reached 0.2 cm^3^, mice were randomized into four groups. sgIF loaded HLC9-EVs were administered intravenously (7.5 mg/kg) along with sorafenib which was given intraperitoneally(100 mg/kg), twice with an interval of 3 days. HLC9-EVs alone were used as control. The size of tumor was measured every 4 days post first injection. Ultimately, all the mice were euthanized and tumors were excised. All animal experimental procedures were performed according to protocols approved by the Animal Care and Use Committee of Southeast University (Nanjing, China).

### Statistical analysis

Data are presented as the mean ± standard deviation (SD) from at least three independent experiments. Student’s t- test and two-way ANOVA (Additional file 1: Table S3–S7) were used to evaluate the significance among different treatment groups, p < 0.05 was considered to be significant.

## Supplementary Information

**Additional file 1: Table S1.** Primers used in this study. **Table S2.** Antibodies used in this study. **Table S3.** Multiple comparisons for CD133 expression in different groups by two-way ANOVA analysis. **Table S4.** Multiple comparisons for protein in different groups in Huh7 cells by two-way ANOVA analysis. **Table S5.** Multiple comparisons for protein in different groups in CD133^+^ Huh7 cells by two-way ANOVA analysis. **Table S6.** Multiple comparisons for protein in different groups in CD133^-^ Huh7 cells by two-way ANOVA analysis. **Table S7.** Multiple comparisons for protein in different groups in excised tumor tissue by two-way ANOVA analysis. **Figure S1.** Schema of plasmids for engineering Cas9/HEK293 cells. Plasmid for obtainLC9-293 cells andHN3LC9-293 cells.Sequences of LAMP2 Signal peptide, HN3, Linker and LAMP2 frame. **Figure S2.** Immunogenicity of EVs. Levels of IFN-γ/TNF-α and IL6 were evaluatedand quantifiedin PBMCs using Elispot. LPS, a lipopolysaccharide, at a concentration of 50 ng/mL was used as a positive control to induce inflammatory factors production. n=3.Histopathological analysis of heart, liver, spleen, lung and kidney sections stained with hematoxylin and eosin of BALB/c mice post-intravenous injection of 10 mg/kg LC9-EVs/HLC9-EVs/PBS thrice with an interval of 2 days. Images were obtained under Leica microscope. Scale bar: 100 μm. **Figure S3.** Cellular uptake of EVs by unsorted and CD133-sorted Huh7 cells.Cellular internalization of both EVs in vitro was viewed with confocal.FACS analysis exhibited in vitro cellular uptake rate of the DiD-, labeled EVs at 3 h post-treatment.Cellular internalization of both EVsby CD133^+/-^ Huh7 cells was viewed with confocal. Scale bar: 1 μm.GPC3 expression on CD133^+/-^ Huh7 and Huh7 cells was analysis by western blotting. **Figure S4.** Validation of sgIF loading efficiency.DNA was visualized after DNase I treatment using agarose gel electrophoresis.Concentrations of DNA within EVs were isolated and measured with and without electroporation. **Figure S5.** Combined therapy of EVs -mediated IQGAP1/FOXM1 destruction with sorafenib. Apoptosis was evaluated through Annexin V/PI staining kit. **Figure S6.** Knockout of IQGAP1/FOXM1 upgrade sorafenib therapeutic effect in CD133^-^ Huh7 cells.PI3K/Akt and MAPK/ERK signaling related protein expression level were examined using western blotting andcorresponding quantitative protein expression results.Nuclear localization of β-catenin was analyzed with immunostaining. Scale bar: 1 μm. Data are expressed as mean ± SD. n=3; *p<0.05, ***p<0.001, by two-way ANOVA.

## Data Availability

Data available within the article or its supplementary materials.
