# Supplementary material for: Engineered extracellular vesicles mediated CRISPR-induced deficiency of IQGAP1/FOXM1 reverses sorafenib resistance in HCC by suppressing cancer stem cells
Source: J Nanobiotechnology. 2023 May 18;21:154. doi: 10.1186/s12951-023-01902-6 (PMC10193671; doi:10.1186/s12951-023-01902-6)
Supplement: Supplementary file 1 — Additional file 1: Table S1. Primers used in this study. Table S2. Antibodies used in this study. Table S3. Multiple comparisons for CD133 expression in different groups by two-way ANOVA analysis. Table S4. Multiple comparisons for protein in different groups in Huh7 cells by two-way ANOVA analysis. Table S5. Multiple comparisons for protein in different groups in CD133+ Huh7 cells by two-way ANOVA analysis. Table S6. Multiple comparisons for protein in different groups in CD133- Huh7 cells by two-way ANOVA analysis. Table S7. Multiple comparisons for protein in different groups in excised tumor tissue by two-way ANOVA analysis. Figure S1. Schema of plasmids for engineering Cas9/HEK293 cells. Plasmid for obtainLC9-293 cells andHN3LC9-293 cells.Sequences of LAMP2 Signal peptide, HN3, Linker and LAMP2 frame. Figure S2. Immunogenicity of EVs. Levels of IFN-γ/TNF-α and IL6 were evaluatedand quantifiedin PBMCs using Elispot. LPS, a lipopolysaccharide, at a concentration of 50 ng/mL was used as a positive control to induce inflammatory factors production. n=3.Histopathological analysis of heart, liver, spleen, lung and kidney sections stained with hematoxylin and eosin of BALB/c mice post-intravenous injection of 10 mg/kg LC9-EVs/HLC9-EVs/PBS thrice with an interval of 2 days. Images were obtained under Leica microscope. Scale bar: 100 μm. Figure S3. Cellular uptake of EVs by unsorted and CD133-sorted Huh7 cells.Cellular internalization of both EVs in vitro was viewed with confocal.FACS analysis exhibited in vitro cellular uptake rate of the DiD-, labeled EVs at 3 h post-treatment.Cellular internalization of both EVsby CD133+/- Huh7 cells was viewed with confocal. Scale bar: 1 μm.GPC3 expression on CD133+/- Huh7 and Huh7 cells was analysis by western blotting. Figure S4. Validation of sgIF loading efficiency.DNA was visualized after DNase I treatment using agarose gel electrophoresis.Concentrations of DNA within EVs were isolated and measured with and without electroporat [file 12951_2023_1902_MOESM1_ESM.docx]

**Supplementary Materials**

**Supplementary Methods**

***In vitro and in vivo safety evaluation***

ELISPOT plates (CTL, hT3009Fp) were pre-treated with 70% ethanol, washed with PBS thrice and incubated with IFN-γ/ TNF-α/IL-6 capture solution overnight at 4°C. After washing the plate with PBS, two types of EVs (LC9-EVs and HLC9-EVs, 20ug) and LPS (50 ng/mL, was used as a positive control) solution were added respectively, and the plate was kept in incubator in 37°C for 20 min. Subsequently, PBMCs at the density of 100,000 cells/well in CTL-test medium was added to these plates. After 24h incubation, the plate was washed twice with 0.05% Tween-PBS, followed by incubation with anti-human IFN-γ/ TNF-α/IL-6 detection solution in room temperature for 2h. Thereafter, the plate was washed thrice with 0.05% Tween-PBS, to which tertiary solution was added. After one hour incubation, the plate was completely wash with distilled water and air dried. After drying, the spots were scanned and counted by CTL S6 Analyzer (CTL, USA).

Nine female BALB/c mice were randomly divided into three groups. Two groups were injected with two formulations of EVs (LC9-EVs and HLC9-EVs) intravenously (10 mg/kg) thrice with an interval of 2 days, respectively. And the other group was treated with PBS as control. The major organ tissues (such as heart, lung, liver, spleen and kidney) were collected at 24 h after the last administration for histochemistry analysis. Then the collected organs were fixed with paraformaldehyde, embedded in paraffin, sectioned, processed for routine hematoxylin and eosin (H&E) staining, and visualized under a Leica microscope.

**Supplementary Tables**

**Table S1. Primers used in this study**

| Primer | Sequence (5’ – 3’) | Use |
| --- | --- | --- |
| C9 Cut M1 | F - GCCGCCGCGGAATTCATGGATAAGAAATACTCAATA GGACTG  R - CTTATCCATGAATTCCGCGGCGGCAGATCTCCTCGG  TACCGG | To create EcoRI site before Cas9 in pCas-guide-GFP to prepare Cas9^-^/sgRNA vector |
| C9 Cut M2 | F - CAGCTGGGAGAATTCCCCAAGAAAAAACGCAAGGTG  R - TTTCTTGGGGAATTCTCCCAGCTGACTCAAATCAAT | To create EcoRI site after Cas9 in pCas-guide-GFP to prepare Cas9^-^/sgRNA vector |
| Eco Del 1 C9 | F - GGGCGGCCGGGCATTCGTCGACTGGAACCGGTACCG  AGG  R - TCGACGAATGCCCGGCCGCCCTATAGTGAGTCGTAT  TAC | To delete EcoRI site in pCas-guide-GFP |
| Eco Del 2 C9 | F - GCAGTTAACGCATTCCCCAGTGGAAAGACGCGCAGG  CAA  R - ACTGGGGAATGCGTTAACTGCCATCCAGCTGATATC  CCC | To delete EcoRI site in pCas-guide-GFP |
| sgFOXM1.1 | F - GATCGGACCTCATCTCCGTCCCCTTG  R - AAAACAAGGGGACGGAGATGAGGTCC | To clone sgFOXM1.1 sequences into pCas-guide-GFP and Cas9^-^/sgRNA to prepare Cas9/sgFOXM1.1 and Cas9^-^/sgFOXM1.1 (sgFOXM1.1) |
| sgFOXM1.2 | F - GATCGTGACCCCTTGCCTGACCCCCG  R - AAAACGGGGGTCAGGCAAGGGGTCAC | To clone sgIQ 1.2 sequences into pCas-guide-GFP to prepare Cas9/sgFOXM1.2 and Cas9^-^/sgFOXM1.2 (sgFOXM1.2) |
| FOXM1  Genome | F - GAGCTCCCGTTCCCAGCAGAC  R - GGCAGCAGGGAGCTATGAGGA | To amply genomic DNA fragment of FOXM1 for T7EI Assay |

**Table S2. Antibodies used in this study**

| Name | Cat# |
| --- | --- |
| CD133-PE antibody | 12-1338-45, eBioscience |
| EpCAM primary antibody | ab187372, Abcam |
| CD133 primary antibody | ab216323, Abcam |
| CD90 primary antibody | Ab133350, Abcam |
| ALDH primary antibody | Ab52492, Abcam |
| GAPDH primary antibody | sc-47724, Santa Cruz |
| Flag primary antibody | SAB4200071, Sigma-Aldrich |
| AcGFP primary antibody | TA180011, ORIGENE |
| CD63 primary antibody | sc-5275, Santa cruz |
| CD40 primary antibody | sc13128, Santa cruz |
| ANXA5 primary antibody | RP01180, Abclonal |
| Cytochrome C primary antibody | A13430, Abclonal |
| GPC3 primary antibody | sc-65443, Santa cruz |
| FOXM1 primary antibody | ab207298, Abcam |
| IQGAP1 primary antibody | ab133490, Abcam |
| Caspase3 primary antibody | ab13847, Abcam |
| BCL2 primary antibody | ab32124， Abcam |
| BAX primary antibody | ab32503, Abcam |
| c-Myc primary antibody | ab32072, Abcam |
| CyclinD1 primary antibody | ab40754, Abcam |
| mTOR primary antibody | ab32028, Abcam |
| pAKT primary antibody | ab81283, Abcam |
| AKT primary antibody | ab108202, Abcam |
| PI3K primary antibody | ab191606, Abcam |
| pMEK primary antibody | ab96379, Abcam |
| MEK primary antibody | ab32091, Abcam |
| pERK primary antibody | ab32538, Abcam |
| ERK primary antibody | ab184699, Abcam |
| β-catenin primary antibody | ab68183, Abcam |
| HRP-conjugated anti-rabbit IgG antibody | ab6721, Abcam |
| HRP-conjugated anti-mouse IgG antibody | ab6728，Abcam |
| Alexa Fluor 488 conjugated secondary antibody | A-11034, Invitrogen |
| Alexa Fluor 532 conjugated secondary antibody | A11002, Invitrogen |

**Table S3 Multiple comparisons for CD133 expression in different groups by two-way ANOVA analysis**

| Group | Bonferroni's multiple comparisons test | Significant? | Summary | Adjusted P Value |
| --- | --- | --- | --- | --- |
| Huh7 | i vs. ii | No | ns | >0.99 |
|  | i vs. iii | Yes | *** | <0.001 |
|  | i vs. iv | Yes | ** | 0.004 |
|  | i vs. v | Yes | *** | <0.001 |
|  | ii vs. iii | Yes | *** | <0.001 |
|  | ii vs. iv | Yes | * | 0.02 |
|  | ii vs. v | Yes | *** | <0.001 |
|  | iii vs. iv | No | ns | 0.4 |
|  | iii vs. v | No | ns | >0.99 |
|  | iv vs. v | Yes | * | 0.02 |
| CD133^+^ Huh7 | i vs. ii | No | ns | >0.99 |
|  | i vs. iii | No | ns | >0.99 |
|  | i vs. iv | No | ns | >0.99 |
|  | i vs. v | Yes | *** | <0.001 |
|  | ii vs. iii | No | ns | >0.99 |
|  | ii vs. iv | No | ns | >0.99 |
|  | ii vs. v | Yes | *** | <0.001 |
|  | iii vs. iv | No | ns | >0.99 |
|  | iii vs. v | Yes | *** | <0.001 |
|  | iv vs. v | Yes | *** | <0.001 |

(i: Mock, ii: HLC9-EVs, iii: Sorafenib, iv: HLC9-EVs+sgIF, v: Sorafenib+HLC9-EVs+sgIF)

**Table S4 Multiple comparisons for protein in different groups in Huh7 cells by two-way ANOVA analysis**

| Protein | Bonferroni's multiple comparisons test | Significant? | Summary | Adjusted P Value |
| --- | --- | --- | --- | --- |
| MEK | i vs. ii | No | ns | >0.99 |
|  | i vs. iii | Yes | *** | <0.001 |
|  | i vs. iv | Yes | ** | 0.003 |
|  | i vs. v | Yes | *** | <0.001 |
|  | ii vs. iii | Yes | *** | <0.001 |
|  | ii vs. iv | Yes | * | 0.02 |
|  | ii vs. v | Yes | *** | <0.001 |
|  | iii vs. iv | Yes | *** | <0.001 |
|  | iii vs. v | Yes | *** | <0.001 |
|  | iv vs. v | Yes | *** | <0.001 |
| pMEK/MEK | i vs. ii | No | ns | 0.67 |
|  | i vs. iii | Yes | *** | <0.001 |
|  | i vs. iv | Yes | *** | <0.001 |
|  | i vs. v | Yes | *** | <0.001 |
|  | ii vs. iii | Yes | *** | <0.001 |
|  | ii vs. iv | Yes | *** | <0.001 |
|  | ii vs. v | Yes | *** | <0.001 |
|  | iii vs. iv | Yes | *** | <0.001 |
|  | iii vs. v | Yes | ** | 0.002 |
|  | iv vs. v | Yes | *** | <0.001 |
| ERK | i vs. ii | No | ns | >0.99 |
|  | i vs. iii | Yes | *** | <0.001 |
|  | i vs. iv | Yes | *** | <0.001 |
|  | i vs. v | Yes | *** | <0.001 |
|  | ii vs. iii | Yes | *** | <0.001 |
|  | ii vs. iv | Yes | *** | <0.001 |
|  | ii vs. v | Yes | *** | <0.001 |
|  | iii vs. iv | No | ns | >0.99 |
|  | iii vs. v | No | ns | 0.08 |
|  | iv vs. v | No | ns | >0.99 |
| pERK/ERK | i vs. ii | No | ns | 0.81 |
|  | i vs. iii | Yes | ** | 0.004 |
|  | i vs. iv | No | ns | >0.99 |
|  | i vs. v | Yes | *** | <0.001 |
|  | ii vs. iii | No | ns | 0.45 |
|  | ii vs. iv | No | ns | 0.12 |
|  | ii vs. v | Yes | *** | <0.001 |
|  | iii vs. iv | Yes | *** | <0.001 |
|  | iii vs. v | Yes | * | 0.03 |
|  | iv vs. v | Yes | *** | <0.001 |
| PI3K | i vs. ii | No | ns | 0.32 |
|  | i vs. iii | Yes | ** | 0.004 |
|  | i vs. iv | Yes | * | 0.02 |
|  | i vs. v | Yes | *** | <0.001 |
|  | ii vs. iii | Yes | *** | <0.001 |
|  | ii vs. iv | Yes | *** | <0.001 |
|  | ii vs. v | Yes | *** | <0.001 |
|  | iii vs. iv | No | ns | >0.99 |
|  | iii vs. v | Yes | *** | <0.001 |
|  | iv vs. v | Yes | *** | <0.001 |
| Akt | i vs. ii | No | ns | 0.1 |
|  | i vs. iii | Yes | *** | <0.001 |
|  | i vs. iv | Yes | *** | <0.001 |
|  | i vs. v | Yes | *** | <0.001 |
|  | ii vs. iii | Yes | *** | <0.001 |
|  | ii vs. iv | Yes | *** | <0.001 |
|  | ii vs. v | Yes | *** | <0.001 |
|  | iii vs. iv | No | ns | 0.13 |
|  | iii vs. v | Yes | *** | <0.001 |
|  | iv vs. v | Yes | *** | <0.001 |
| pAKT/AKT | i vs. ii | No | ns | 0.13 |
|  | i vs. iii | Yes | *** | <0.001 |
|  | i vs. iv | No | ns | 0.61 |
|  | i vs. v | Yes | *** | <0.001 |
|  | ii vs. iii | Yes | *** | <0.001 |
|  | ii vs. iv | Yes | * | 0.04 |
|  | ii vs. v | Yes | *** | <0.001 |
|  | iii vs. iv | Yes | *** | <0.001 |
|  | iii vs. v | No | ns | 0.36 |
|  | iv vs. v | Yes | *** | <0.001 |
| mTOR | i vs. ii | No | ns | 0.1 |
|  | i vs. iii | No | ns | 0.09 |
|  | i vs. iv | No | ns | >0.99 |
|  | i vs. v | Yes | *** | <0.001 |
|  | ii vs. iii | Yes | *** | <0.001 |
|  | ii vs. iv | Yes | * | 0.01 |
|  | ii vs. v | Yes | *** | <0.001 |
|  | iii vs. iv | No | ns | 0.15 |
|  | iii vs. v | Yes | *** | <0.001 |
|  | iv vs. v | Yes | *** | <0.001 |
| Cylin D1 | i vs. ii | No | ns | 0.33 |
|  | i vs. iii | Yes | *** | <0.001 |
|  | i vs. iv | Yes | *** | <0.001 |
|  | i vs. v | Yes | *** | <0.001 |
|  | ii vs. iii | Yes | *** | <0.001 |
|  | ii vs. iv | Yes | *** | <0.001 |
|  | ii vs. v | Yes | *** | <0.001 |
|  | iii vs. iv | Yes | *** | <0.001 |
|  | iii vs. v | No | ns | 0.06 |
|  | iv vs. v | Yes | *** | <0.001 |
| c-Myc | i vs. ii | No | ns | 0.62 |
|  | i vs. iii | Yes | * | 0.04 |
|  | i vs. iv | No | ns | 0.06 |
|  | i vs. v | Yes | *** | <0.001 |
|  | ii vs. iii | Yes | *** | <0.001 |
|  | ii vs. iv | No | ns | >0.99 |
|  | ii vs. v | Yes | *** | <0.001 |
|  | iii vs. iv | Yes | *** | <0.001 |
|  | iii vs. v | Yes | *** | <0.001 |
|  | iv vs. v | Yes | *** | <0.001 |

(i: Mock, ii: HLC9-EVs, iii: Sorafenib, iv: HLC9-EVs+sgIF, v: Sorafenib+HLC9-EVs+sgIF)

**Table S5 Multiple comparisons for protein in different groups in CD133^+^ Huh7 cells by two-way ANOVA analysis**

| Protein | Bonferroni's multiple comparisons test | Significant? | Summary | Adjusted P Value |
| --- | --- | --- | --- | --- |
| MEK | i vs. ii | No | ns | >0.99 |
|  | i vs. iii | No | ns | >0.99 |
|  | i vs. iv | No | ns | >0.99 |
|  | i vs. v | Yes | *** | <0.001 |
|  | ii vs. iii | No | ns | 0.49 |
|  | ii vs. iv | No | ns | >0.99 |
|  | ii vs. v | Yes | *** | <0.001 |
|  | iii vs. iv | No | ns | >0.99 |
|  | iii vs. v | No | ns | 0.07 |
|  | iv vs. v | Yes | ** | 0.008 |
| pMEK/MEK | i vs. ii | No | ns | >0.99 |
|  | i vs. iii | No | ns | >0.99 |
|  | i vs. iv | No | ns | >0.99 |
|  | i vs. v | No | ns | >0.99 |
|  | ii vs. iii | No | ns | >0.99 |
|  | ii vs. iv | No | ns | >0.99 |
|  | ii vs. v | No | ns | >0.99 |
|  | iii vs. iv | No | ns | >0.99 |
|  | iii vs. v | No | ns | >0.99 |
|  | iv vs. v | No | ns | >0.99 |
| ERK | i vs. ii | No | ns | >0.99 |
|  | i vs. iii | No | ns | >0.99 |
|  | i vs. iv | No | ns | >0.99 |
|  | i vs. v | No | ns | 0.53 |
|  | ii vs. iii | No | ns | 0.2 |
|  | ii vs. iv | No | ns | >0.99 |
|  | ii vs. v | Yes | * | 0.04 |
|  | iii vs. iv | No | ns | >0.99 |
|  | iii vs. v | No | ns | >0.99 |
|  | iv vs. v | No | ns | >0.99 |
| pERK/ERK | i vs. ii | No | ns | >0.99 |
|  | i vs. iii | No | ns | >0.99 |
|  | i vs. iv | No | ns | 0.91 |
|  | i vs. v | No | ns | >0.99 |
|  | ii vs. iii | No | ns | >0.99 |
|  | ii vs. iv | No | ns | >0.99 |
|  | ii vs. v | No | ns | >0.99 |
|  | iii vs. iv | No | ns | 0.86 |
|  | iii vs. v | No | ns | >0.99 |
|  | iv vs. v | No | ns | 0.74 |
| PI3K | i vs. ii | No | ns | >0.99 |
|  | i vs. iii | Yes | ** | 0.009 |
|  | i vs. iv | No | ns | >0.99 |
|  | i vs. v | Yes | *** | <0.001 |
|  | ii vs. iii | Yes | ** | 0.002 |
|  | ii vs. iv | No | ns | 0.81 |
|  | ii vs. v | Yes | *** | <0.001 |
|  | iii vs. iv | No | ns | 0.24 |
|  | iii vs. v | No | ns | >0.99 |
|  | iv vs. v | Yes | * | 0.01 |
| Akt | i vs. ii | No | ns | >0.99 |
|  | i vs. iii | No | ns | 0.05 |
|  | i vs. iv | No | ns | >0.99 |
|  | i vs. v | Yes | *** | <0.001 |
|  | ii vs. iii | Yes | ** | 0.002 |
|  | ii vs. iv | No | ns | >0.99 |
|  | ii vs. v | Yes | *** | <0.001 |
|  | iii vs. iv | Yes | * | 0.01 |
|  | iii vs. v | No | ns | >0.99 |
|  | iv vs. v | Yes | *** | <0.001 |
| pAKT/AKT | i vs. ii | No | ns | 0.36 |
|  | i vs. iii | Yes | *** | <0.001 |
|  | i vs. iv | No | ns | 0.64 |
|  | i vs. v | Yes | *** | <0.001 |
|  | ii vs. iii | Yes | *** | <0.001 |
|  | ii vs. iv | No | ns | >0.99 |
|  | ii vs. v | Yes | *** | <0.001 |
|  | iii vs. iv | Yes | *** | <0.001 |
|  | iii vs. v | Yes | *** | <0.001 |
|  | iv vs. v | Yes | *** | <0.001 |
| mTOR | i vs. ii | No | ns | >0.99 |
|  | i vs. iii | No | ns | 0.07 |
|  | i vs. iv | No | ns | >0.99 |
|  | i vs. v | Yes | ** | 0.003 |
|  | ii vs. iii | Yes | *** | <0.001 |
|  | ii vs. iv | No | ns | >0.99 |
|  | ii vs. v | Yes | *** | <0.001 |
|  | iii vs. iv | Yes | ** | 0.007 |
|  | iii vs. v | No | ns | >0.99 |
|  | iv vs. v | Yes | *** | <0.001 |
| cylin D1 | i vs. ii | No | ns | >0.99 |
|  | i vs. iii | Yes | * | 0.01 |
|  | i vs. iv | No | ns | >0.99 |
|  | i vs. v | Yes | ** | 0.002 |
|  | ii vs. iii | Yes | ** | 0.003 |
|  | ii vs. iv | No | ns | >0.99 |
|  | ii vs. v | Yes | *** | <0.001 |
|  | iii vs. iv | No | ns | 0.07 |
|  | iii vs. v | No | ns | >0.99 |
|  | iv vs. v | Yes | * | 0.02 |
| c-Myc | i vs. ii | No | ns | 0.59 |
|  | i vs. iii | Yes | * | 0.02 |
|  | i vs. iv | No | ns | >0.99 |
|  | i vs. v | Yes | *** | <0.001 |
|  | ii vs. iii | Yes | *** | <0.001 |
|  | ii vs. iv | Yes | * | 0.02 |
|  | ii vs. v | Yes | *** | <0.001 |
|  | iii vs. iv | No | ns | 0.48 |
|  | iii vs. v | No | ns | 0.16 |
|  | iv vs. v | Yes | ** | 0.001 |

(i: Mock, ii: HLC9-EVs, iii: Sorafenib, iv: HLC9-EVs+sgIF, v: Sorafenib+HLC9-EVs+sgIF)

**Table S6 Multiple comparisons for protein in different groups in CD133^-^ Huh7 cells by two-way ANOVA analysis**

| Protein | Bonferroni's multiple comparisons test | Significant? | Summary | Adjusted P Value |
| --- | --- | --- | --- | --- |
| MEK | i vs. ii | No | ns | >0.99 |
|  | i vs. iii | Yes | * | 0.01 |
|  | i vs. iv | No | ns | >0.99 |
|  | i vs. v | No | ns | >0.99 |
|  | ii vs. iii | No | ns | 0.71 |
|  | ii vs. iv | No | ns | >0.99 |
|  | ii vs. v | Yes | * | 0.04 |
|  | iii vs. iv | No | ns | 0.34 |
|  | iii vs. v | Yes | *** | <0.001 |
|  | iv vs. v | No | ns | 0.1 |
| pMEK/MEK | i vs. ii | No | ns | >0.99 |
|  | i vs. iii | No | ns | >0.99 |
|  | i vs. iv | No | ns | >0.99 |
|  | i vs. v | No | ns | >0.99 |
|  | ii vs. iii | No | ns | >0.99 |
|  | ii vs. iv | No | ns | >0.99 |
|  | ii vs. v | No | ns | 0.83 |
|  | iii vs. iv | No | ns | >0.99 |
|  | iii vs. v | No | ns | >0.99 |
|  | iv vs. v | No | ns | >0.99 |
| ERK | i vs. ii | No | ns | >0.99 |
|  | i vs. iii | No | ns | 0.21 |
|  | i vs. iv | No | ns | 0.13 |
|  | i vs. v | No | ns | >0.99 |
|  | ii vs. iii | No | ns | 0.07 |
|  | ii vs. iv | Yes | * | 0.04 |
|  | ii vs. v | No | ns | >0.99 |
|  | iii vs. iv | No | ns | >0.99 |
|  | iii vs. v | No | ns | 0.06 |
|  | iv vs. v | Yes | * | 0.03 |
| pERK/ERK | i vs. ii | No | ns | >0.99 |
|  | i vs. iii | Yes | *** | <0.001 |
|  | i vs. iv | No | ns | >0.99 |
|  | i vs. v | No | ns | 0.38 |
|  | ii vs. iii | Yes | *** | <0.001 |
|  | ii vs. iv | No | ns | >0.99 |
|  | ii vs. v | No | ns | 0.31 |
|  | iii vs. iv | Yes | ** | 0.004 |
|  | iii vs. v | No | ns | 0.06 |
|  | iv vs. v | No | ns | >0.99 |
| PI3K | i vs. ii | No | ns | >0.99 |
|  | i vs. iii | No | ns | >0.99 |
|  | i vs. iv | No | ns | >0.99 |
|  | i vs. v | No | ns | >0.99 |
|  | ii vs. iii | No | ns | >0.99 |
|  | ii vs. iv | No | ns | >0.99 |
|  | ii vs. v | No | ns | >0.99 |
|  | iii vs. iv | No | ns | >0.99 |
|  | iii vs. v | No | ns | 0.84 |
|  | iv vs. v | No | ns | >0.99 |
| Akt | i vs. ii | No | ns | >0.99 |
|  | i vs. iii | No | ns | >0.99 |
|  | i vs. iv | No | ns | >0.99 |
|  | i vs. v | No | ns | 0.5 |
|  | ii vs. iii | No | ns | >0.99 |
|  | ii vs. iv | No | ns | >0.99 |
|  | ii vs. v | No | ns | 0.08 |
|  | iii vs. iv | No | ns | >0.99 |
|  | iii vs. v | Yes | * | 0.03 |
|  | iv vs. v | No | ns | 0.49 |
| pAKT/AKT | i vs. ii | No | ns | >0.99 |
|  | i vs. iii | No | ns | >0.99 |
|  | i vs. iv | No | ns | 0.14 |
|  | i vs. v | Yes | *** | <0.001 |
|  | ii vs. iii | No | ns | 0.86 |
|  | ii vs. iv | No | ns | >0.99 |
|  | ii vs. v | Yes | * | 0.01 |
|  | iii vs. iv | Yes | * | 0.02 |
|  | iii vs. v | Yes | *** | <0.001 |
|  | iv vs. v | No | ns | 0.63 |
| mTOR | i vs. ii | No | ns | >0.99 |
|  | i vs. iii | No | ns | >0.99 |
|  | i vs. iv | No | ns | >0.99 |
|  | i vs. v | No | ns | >0.99 |
|  | ii vs. iii | No | ns | >0.99 |
|  | ii vs. iv | No | ns | >0.99 |
|  | ii vs. v | No | ns | >0.99 |
|  | iii vs. iv | No | ns | >0.99 |
|  | iii vs. v | No | ns | >0.99 |
|  | iv vs. v | No | ns | >0.99 |
| cylin D1    cylin D1 | i vs. ii | No | ns | >0.99 |
|  | i vs. iii | No | ns | 0.06 |
|  | i vs. iv | Yes | * | 0.01 |
|  | i vs. v | Yes | *** | <0.001 |
|  | ii vs. iii | No | ns | 0.12 |
|  | ii vs. iv | Yes | * | 0.02 |
|  | ii vs. v | Yes | *** | <0.001 |
|  | iii vs. iv | No | ns | >0.99 |
|  | iii vs. v | Yes | ** | 0.004 |
|  | iv vs. v | Yes | * | 0.02 |
| c-Myc | i vs. ii | No | ns | >0.99 |
|  | i vs. iii | Yes | *** | <0.001 |
|  | i vs. iv | Yes | ** | 0.004 |
|  | i vs. v | Yes | *** | <0.001 |
|  | ii vs. iii | Yes | *** | <0.001 |
|  | ii vs. iv | Yes | ** | 0.002 |
|  | ii vs. v | Yes | *** | <0.001 |
|  | iii vs. iv | No | ns | 0.17 |
|  | iii vs. v | No | ns | >0.99 |
|  | iv vs. v | Yes | * | 0.01 |

(i: Mock, ii: HLC9-EVs, iii: Sorafenib, iv: HLC9-EVs+sgIF, v: Sorafenib+HLC9-EVs+sgIF)

**Table S7 Multiple comparisons for protein in different groups in excised tumor tissue by two-way ANOVA analysis**

| Protein | Bonferroni's multiple comparisons test | Significant? | Summary | Adjusted P Value |
| --- | --- | --- | --- | --- |
| IQGAP1 | i vs. ii | No | ns | >0.99 |
|  | i vs. iii | Yes | ** | 0.004 |
|  | i vs. v | Yes | *** | <0.001 |
|  | ii vs. iii | Yes | * | 0.02 |
|  | ii vs. v | Yes | *** | <0.001 |
|  | iii vs. v | Yes | ** | 0.008 |
| FOXM1 | i vs. ii | No | ns | >0.99 |
|  | i vs. iii | Yes | * | 0.03 |
|  | i vs. v | Yes | *** | <0.001 |
|  | ii vs. iii | No | ns | 0.16 |
|  | ii vs. v | Yes | *** | <0.001 |
|  | iii vs. v | Yes | * | 0.02 |

(i: Mock, ii: HLC9-EVs, iii: Sorafenib, iv: HLC9-EVs+sgIF, v: Sorafenib+HLC9-EVs+sgIF)

**Supplementary Figures**

**Figure S1.**


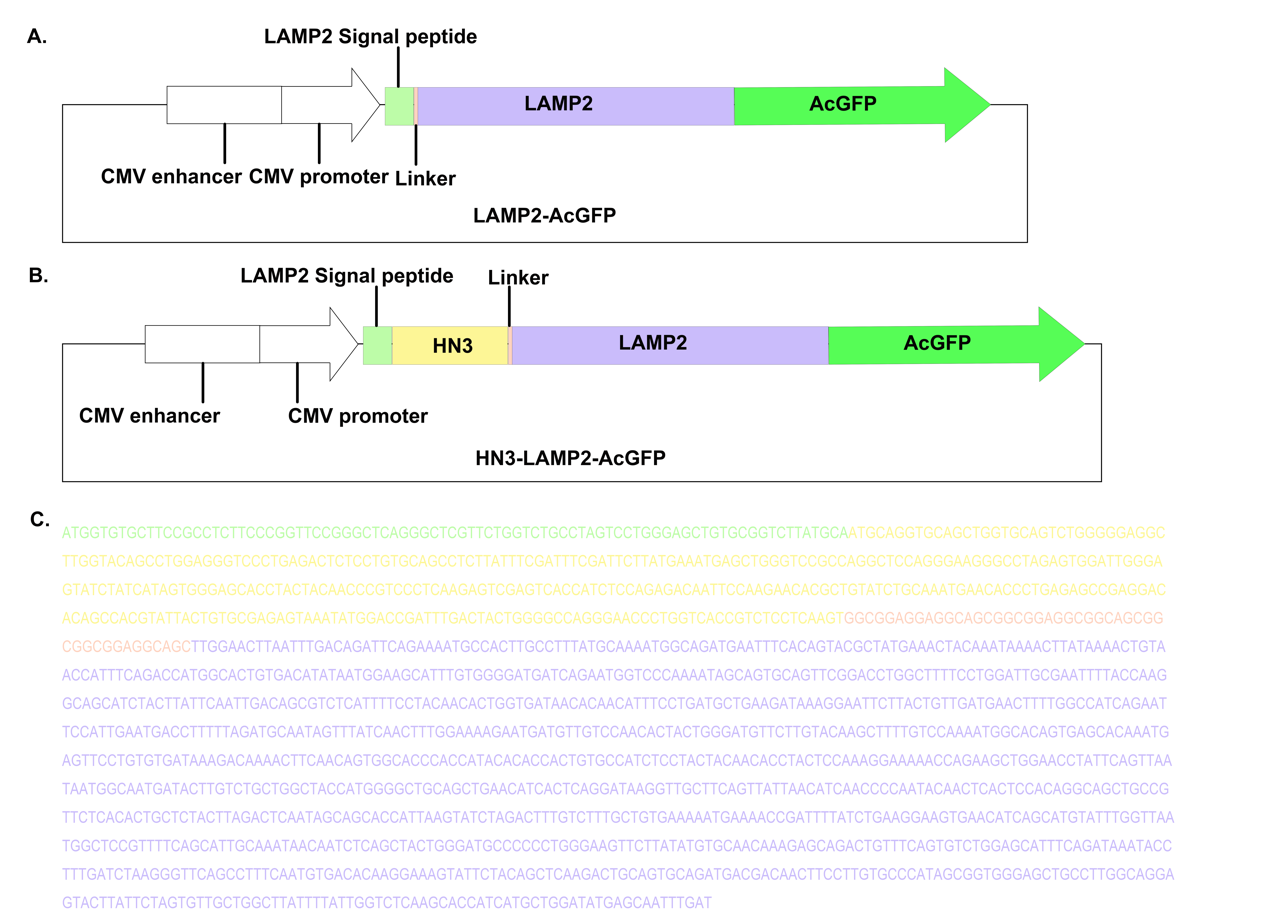


Figure S1 Schema of plasmids for engineering Cas9/HEK293 cells. Plasmid for obtain (A) LC9-293 cells and (B) HN3LC9-293 cells. (C) Sequences of LAMP2 Signal peptide, HN3, Linker and LAMP2 frame.

**Figure S2.**


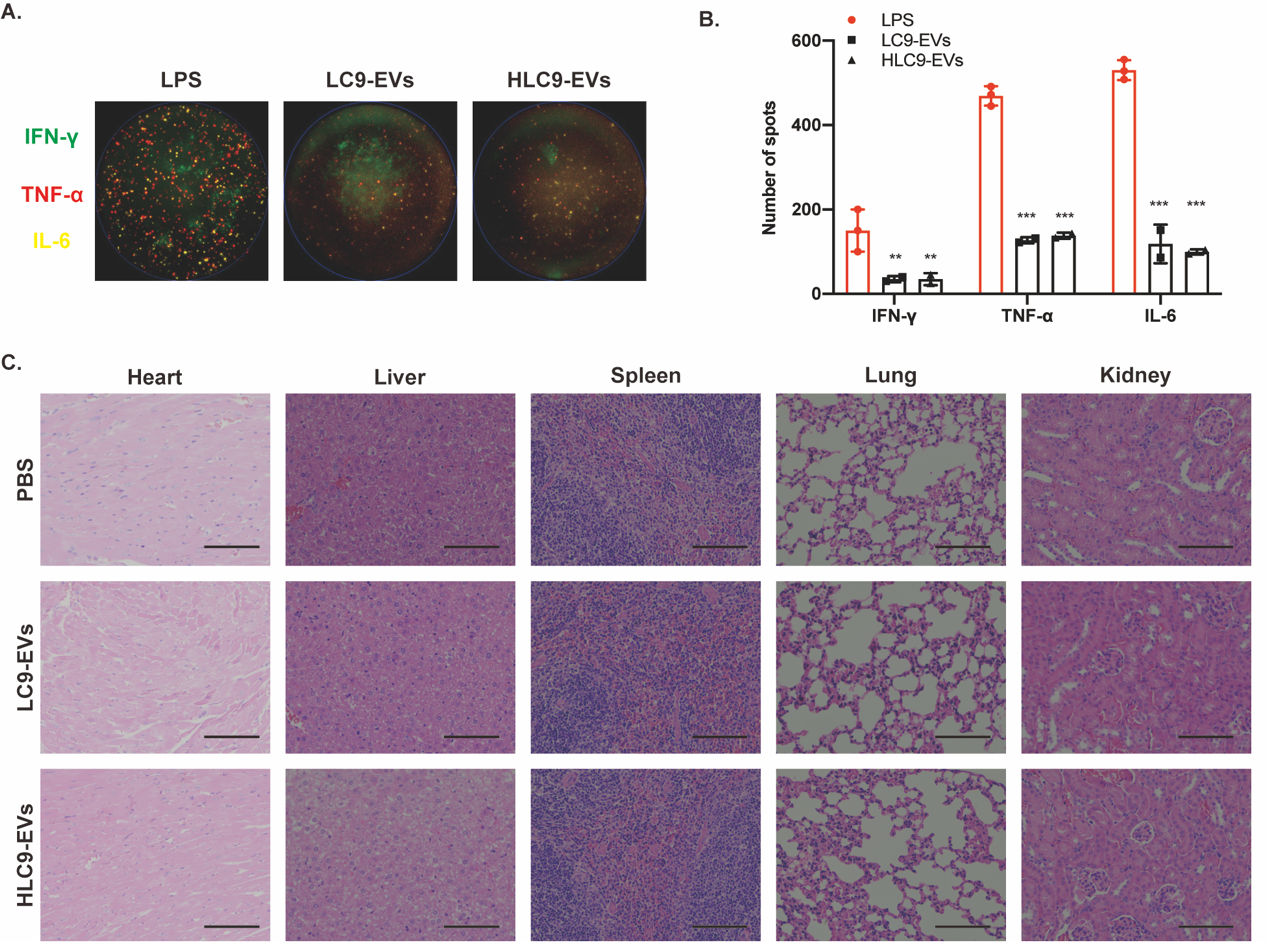


Figure S2 Immunogenicity of EVs. Levels of IFN-γ/TNF-α and IL6 were evaluated (A) and quantified (B) in PBMCs using Elispot. LPS, a lipopolysaccharide, at a concentration of 50 ng/mL was used as a positive control to induce inflammatory factors production. n=3. (C) Histopathological analysis of heart, liver, spleen, lung and kidney sections stained with hematoxylin and eosin of BALB/c mice post-intravenous injection of 10 mg/kg LC9-EVs/HLC9-EVs/PBS thrice with an interval of 2 days. Images were obtained under Leica microscope. Scale bar: 100 μm.

**Figure S3**


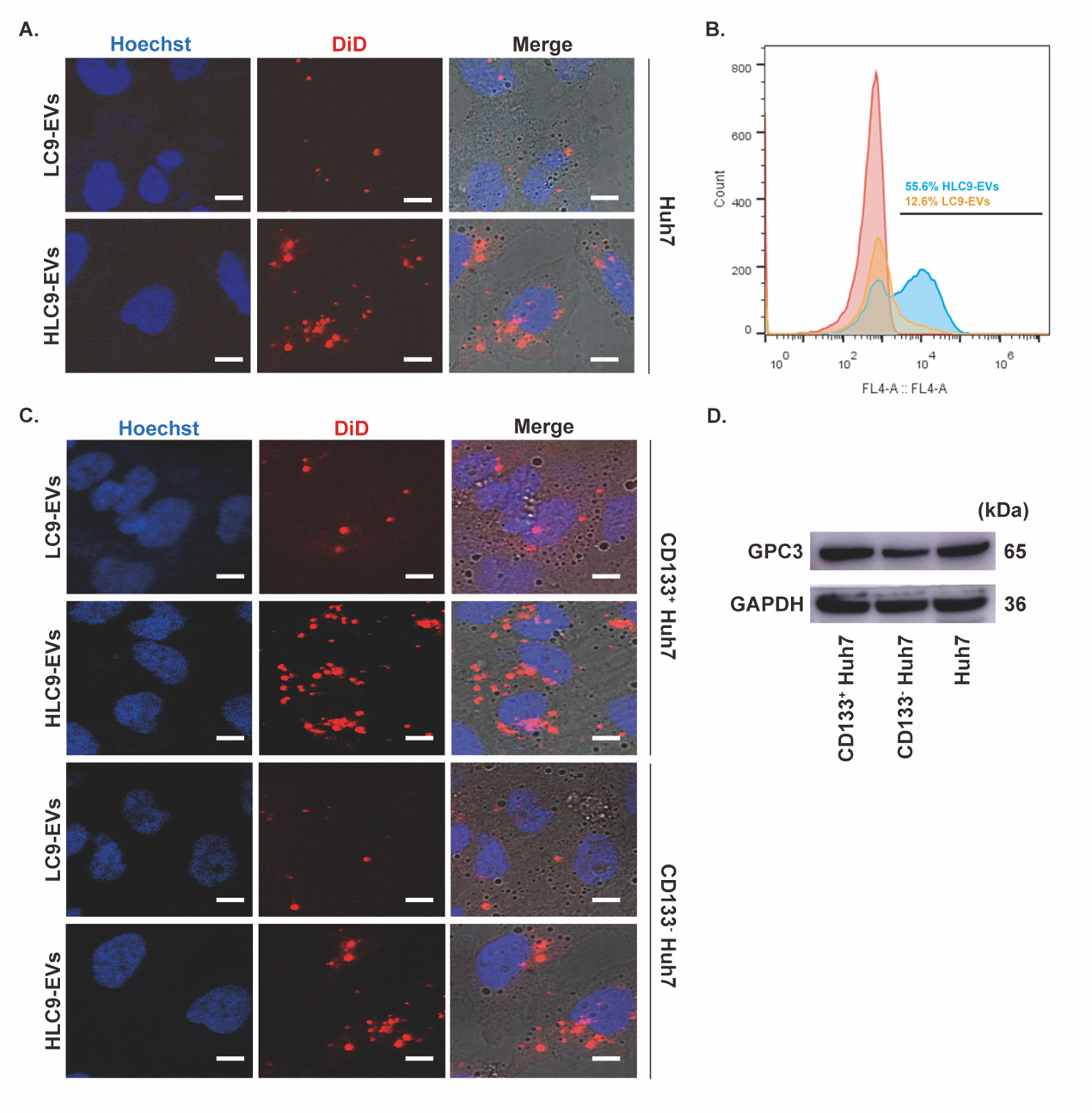


Figure S3 Cellular uptake of EVs by unsorted and CD133-sorted Huh7 cells. (A) Cellular internalization of both EVs (LC9-EVs and HLC9-EVs) *in vitro* was viewed with confocal. (B) FACS analysis exhibited *in vitro* cellular uptake rate of the DiD-(red), labeled EVs at 3 h post-treatment. (C) Cellular internalization of both EVs (LC9-EVs and HLC9-EVs) by CD133^+/-^ Huh7 cells was viewed with confocal. Scale bar: 1 μm. (D)GPC3 expression on CD133^+/-^ Huh7 and Huh7 cells was analysis by western blotting.

**Figure S4**


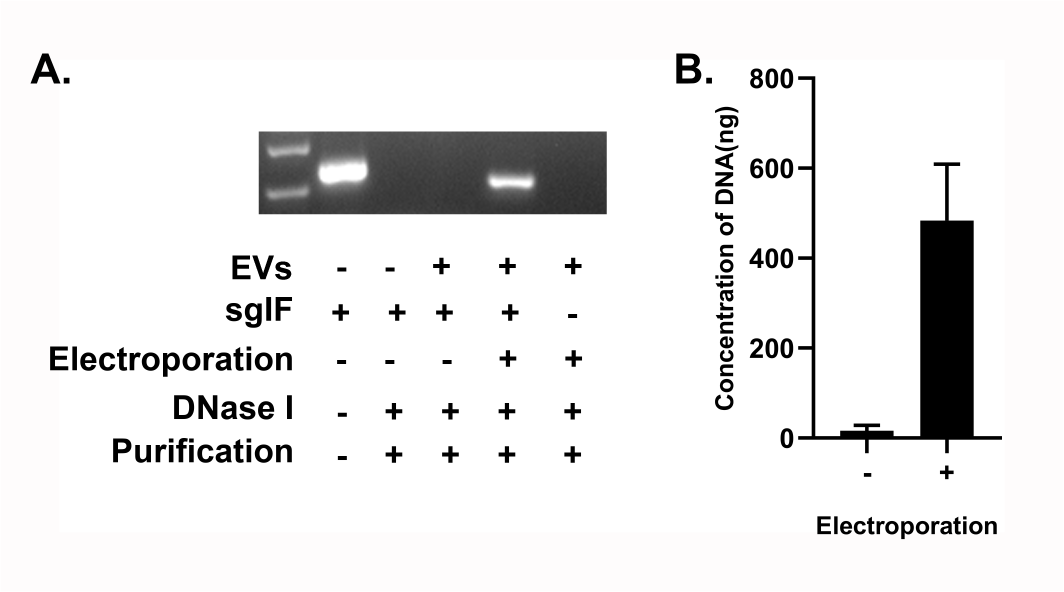


Figure S4 Validation of sgIF loading efficiency. (A) DNA was visualized after DNase I treatment using agarose gel electrophoresis. (B) Concentrations of DNA within EVs were isolated and measured with and without electroporation.

**Figure S5**


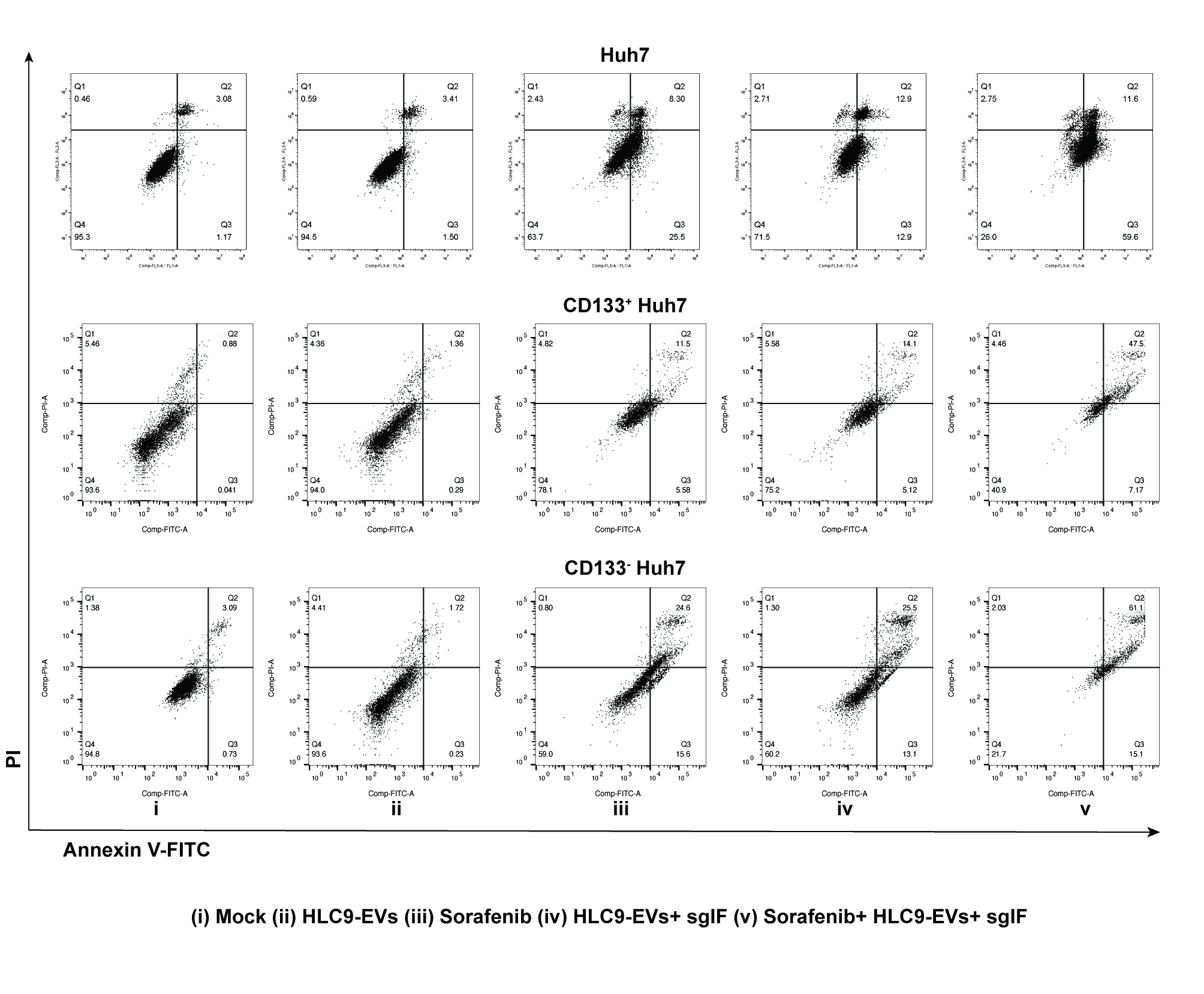


Figure S5 Combined therapy of EVs -mediated IQGAP1/FOXM1 destruction with sorafenib. Apoptosis was evaluated through Annexin V/PI staining kit.

**Figure S6**


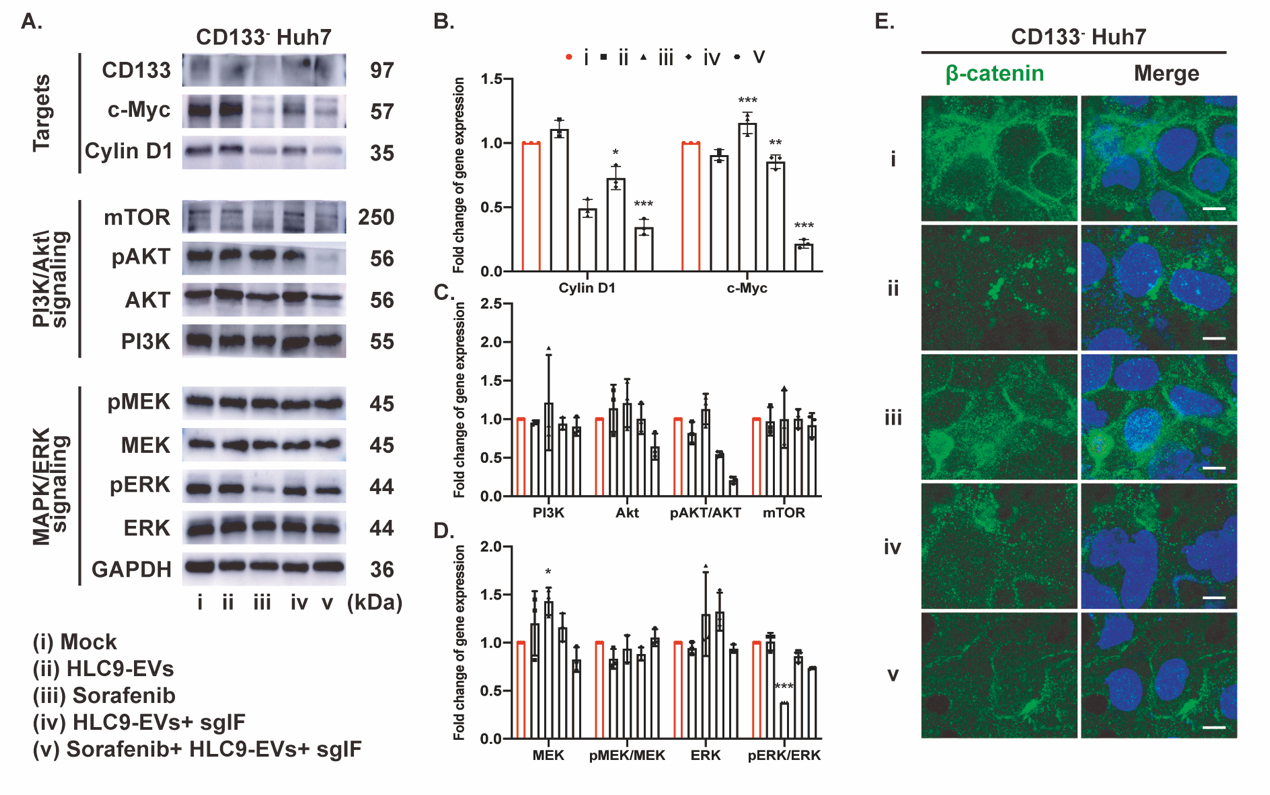


Figure S6 Knockout of IQGAP1/FOXM1 upgrade sorafenib therapeutic effect in CD133^-^ Huh7 cells. (A) PI3K/Akt and MAPK/ERK signaling related protein expression level were examined using western blotting and (B-D) corresponding quantitative protein expression results. (E)Nuclear localization of β-catenin was analyzed with immunostaining. Scale bar: 1 μm. Data are expressed as mean ± SD. n=3; *p<0.05, ***p<0.001, by two-way ANOVA.
